# Supplementary material for: Combined Single-Cell Functional and Gene Expression Analysis Resolves Heterogeneity within Stem Cell Populations
Source: Cell Stem Cell. 2015 Jun 4;16(6):712–24. doi: 10.1016/j.stem.2015.04.004 (PMC4460190; doi:10.1016/j.stem.2015.04.004)
Supplement: Document S1. Supplemental Experimental Procedures, Figures S1–S5, and Table S1 [file mmc1.pdf]

Cell Stem Cell
Supplemental Information

Combined Single-Cell Functional and Gene Expression Analysis Resolves Heterogeneity within Stem Cell Populations

Nicola K. Wilson, David G. Kent, Florian Buettner, Mona Shehata, Iain C. Macaulay, Fernando J. Calero-Nieto, Manuel Sánchez Castillo, Caroline A. Oedekoven, Evangelia Diamanti, Reiner Schulte, Chris P. Ponting, Thierry Voet, Carlos Caldas, John Stingl, Anthony R. Green, Fabian J. Theis, and Berthold Göttgens

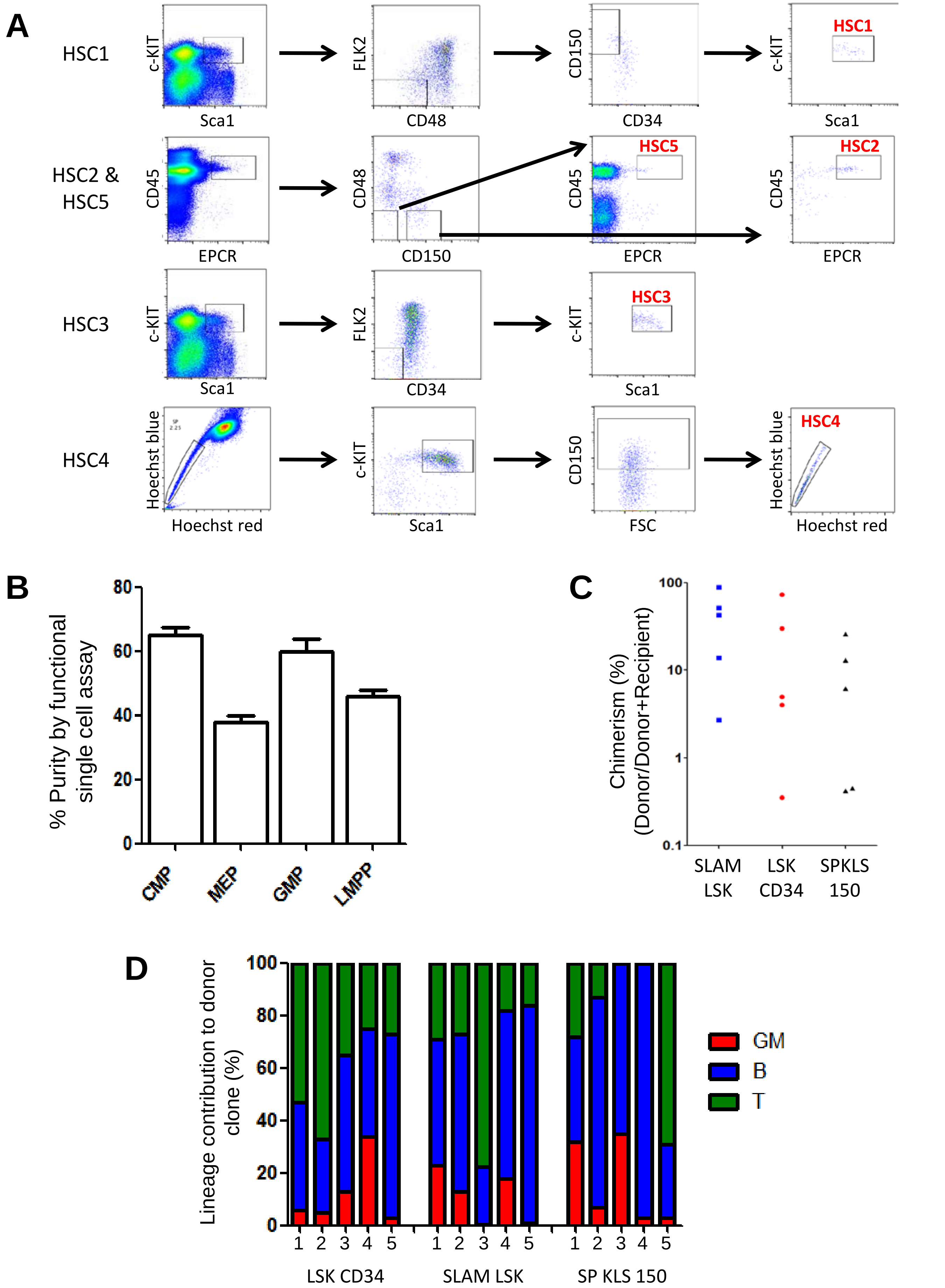

Wilson et al Figure S1

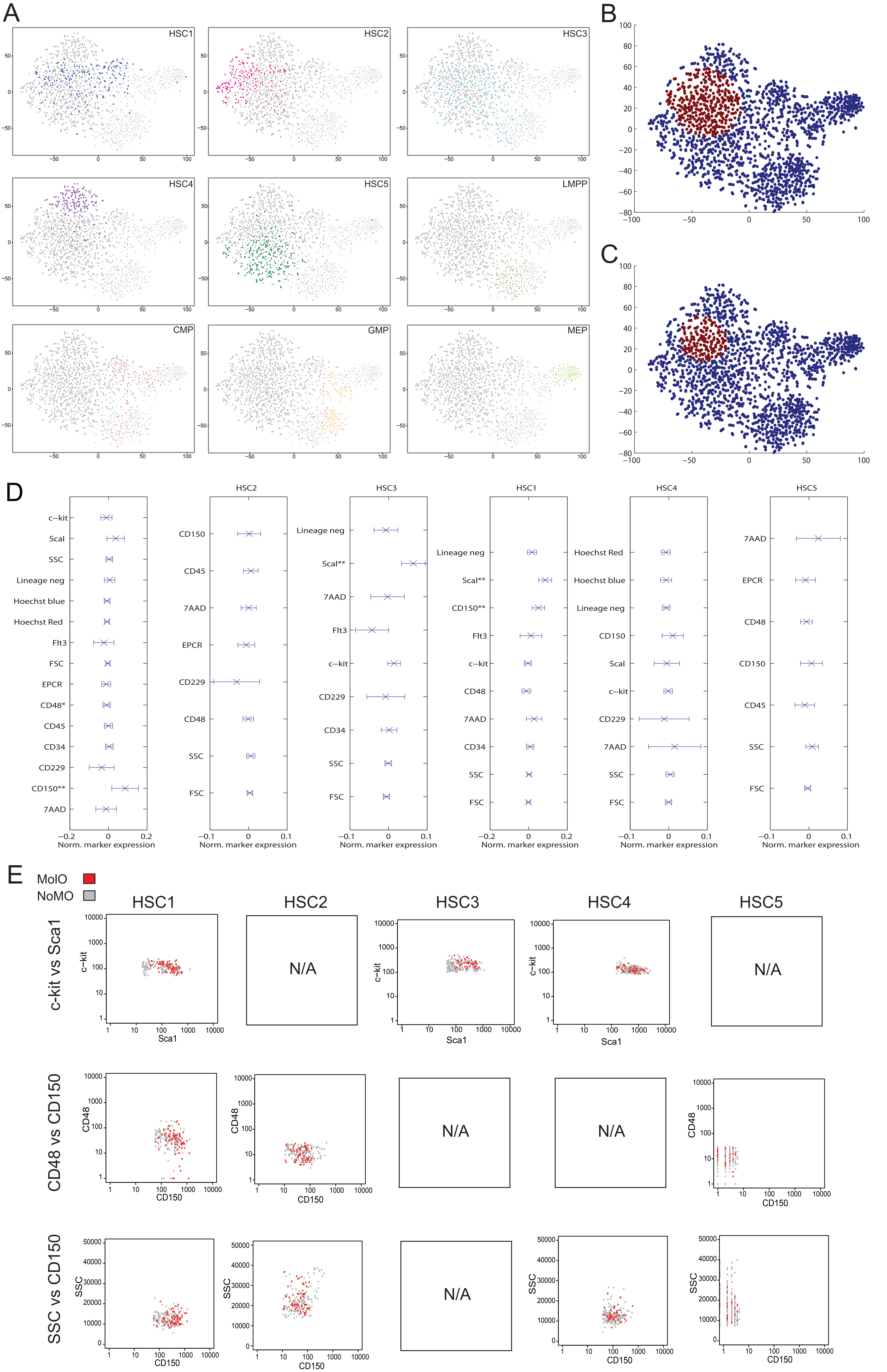

Wilson et al Figure S2

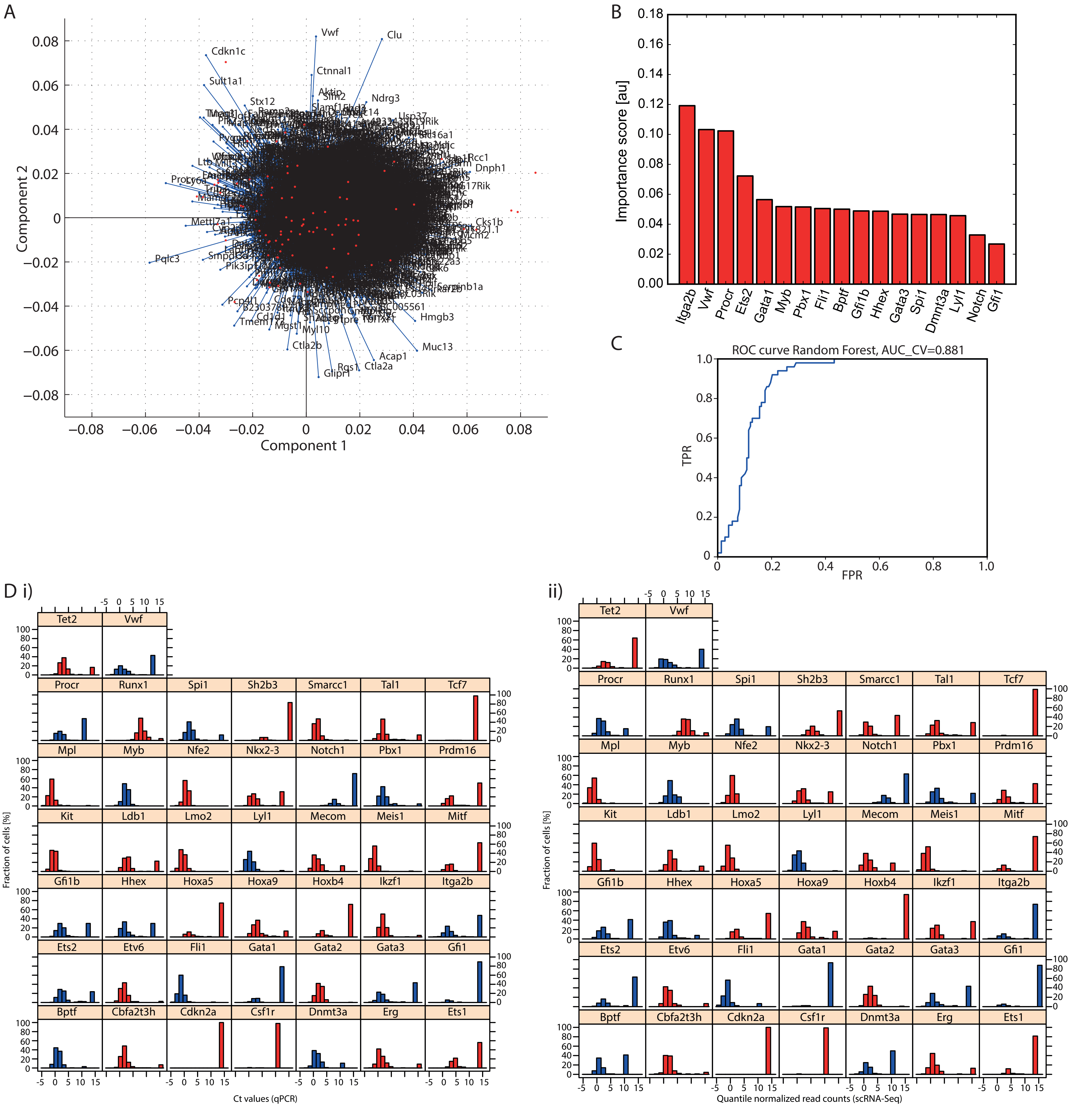

Wilson et al Figure S3

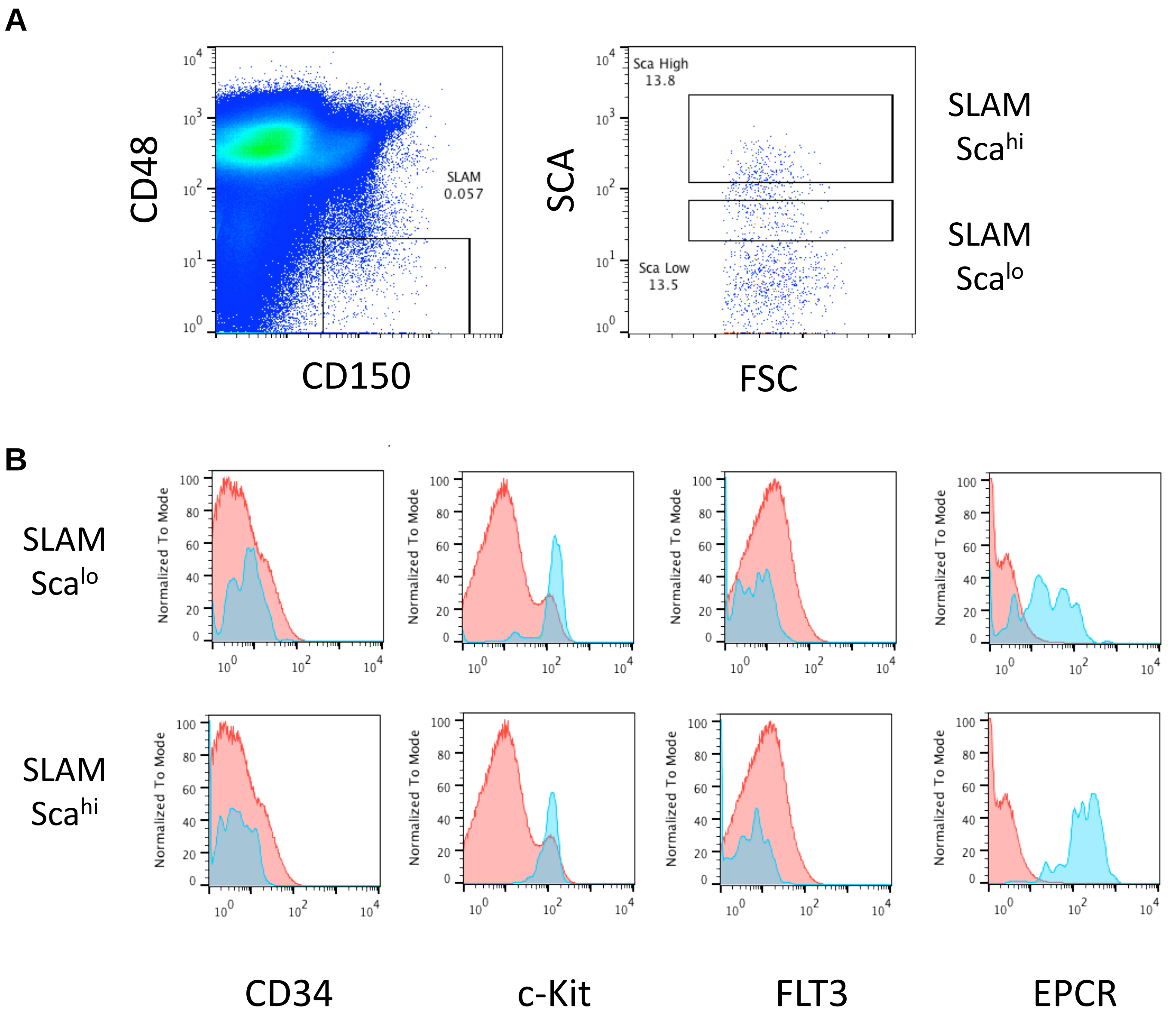

Wilson et al Figure S4

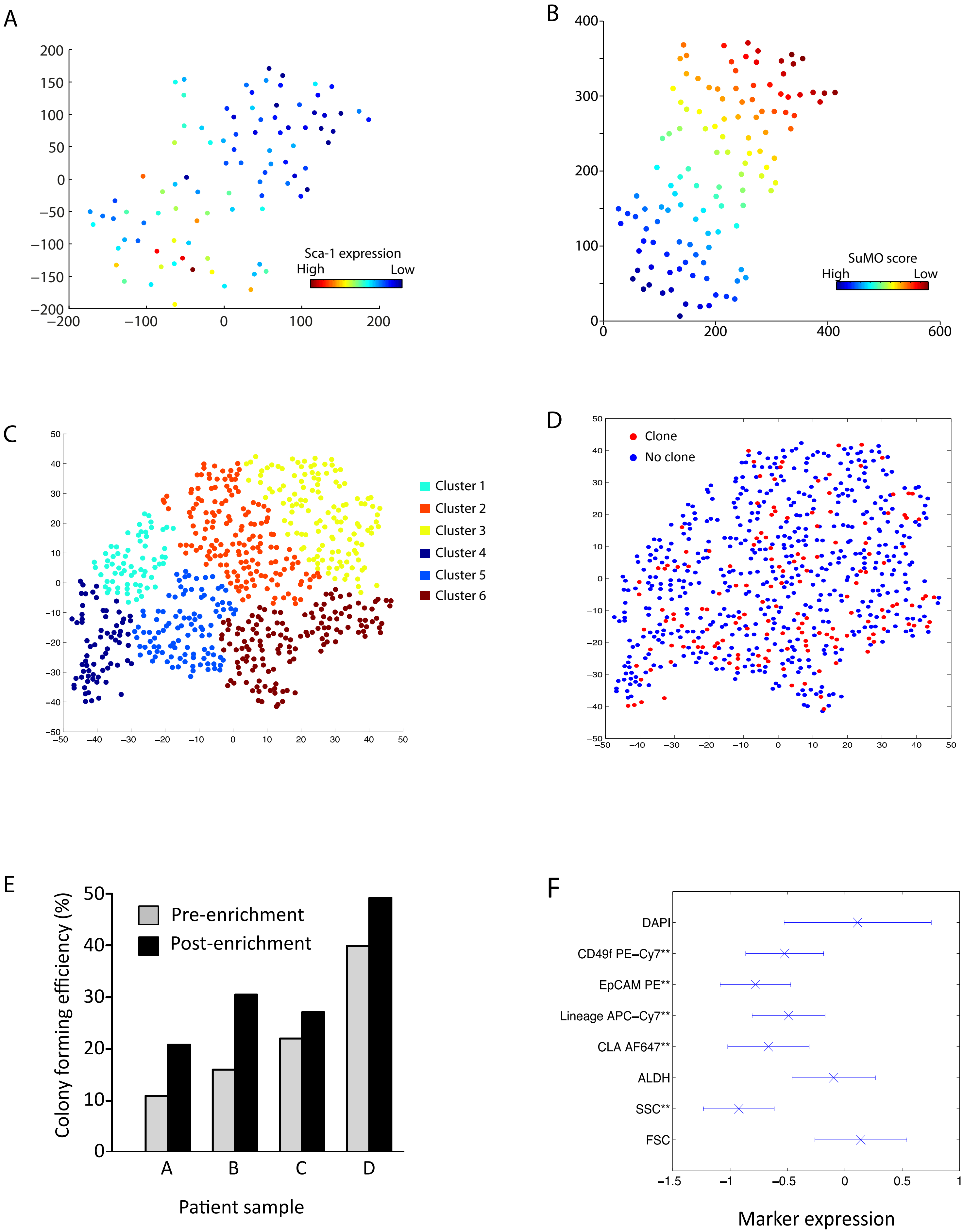

Wilson et al Figure S5

Supplemental Information

Figure S1, related to Figure 1; Functional validation of sorted populations for gene expression profiling.

A) Sorting and gating strategies for the isolation of all HSC populations. B) Individual progenitor cells were isolated and seeded in a methylcellulose-based colony assay. For CMP, GMP and MEPs, 500 cells were isolated and placed into methylcellulose cultures. Colonies were morphologically scored as mixed, erythroid, or granulocytic/monocytic and the percentage of the correct colony type (e.g., granulocyte colony for GMP) is depicted. For LMPPs, single cells were sorted onto OP9 feeder cells and scored for the presence of myeloid and lymphoid elements. The percentage purity is indicated on the y axis. Error bars represent data ± SEM. C) Each individual dot represents the donor chimerism at 16 weeks in a recipient of 10 phenotypically identified HSCs. The phenotypes are indicated on the x-axis and the % donor contribution on the y-axis. D) The stacked bar graphs depict the relative contribution of the donor clone to each of the granulocyte/monocyte (GM, red), B-cell (B, blue), and T-cell (T, green) lineages.

Figure S2, related to Figure 2; Robustness analysis.

t-SNE plot of all cells calculated from the 43 genes analysed by Fluidigm. Axes are in arbitrary units. A) Individual populations are shown for clarity. B-C) t-SNE plot with the MolO HSCs identified by the computational weighting highlighted in red. B) Leeway factor of 70%. C) Leeway factor of 90%. D) Difference in normalised marker expression between MolO cells and NoMO cells for each sorting strategy. Error bars correspond to 1 standard deviation. First column represents all MolO cells compared to all other HSCs across sorting

strategies. * = p<0.05, ** = p<0.01. E) FACs plots for the individual HSC sorting strategies, showing c-kit, Sca-1, CD48 and CD150. MolO HSCs are highlighted in red, NoMO HSCs are highlighted in gray.

Figure S3, related to Figure 3; RNA-Seq analysis

A) Principal component loading plot, indicating the 4533 differentially regulated genes identified from the scRNA-Seq. The 92 single cells which were analysed by scRNA-Seq are shown within the plot as red circles. B) Importance of the individual genes for predicting MolO cells using random forests, quantified as the total decrease in node impurity averaged over all trees in the random forest (gini importance). C) ROC curve quantifying classification performance and generalizability of MolO predictor using 10-fold cross-validation. An area under the cross-validated ROC curve of 0.881 indicates that in 88% percent of predictions, a randomly drawn MolO cell would receive a higher MolO score than a randomly drawn NoMO cell. D) Distribution plots are shown for both the qPCR and quantile normalized RNA-Seq read counts. Only genes assayed by qPCR are shown. Heterogeneous genes are shown in blue. i) qPCR. ii) Normalised RNA-Seq read counts.

Figure S4, related to Figure 4; SLAM Sca^{lo} and SLAM Sca^{hi} populations predominantly express other HSC markers.

A) Sorting strategy used to isolate SLAM Sca^{lo} and SLAM Sca^{hi} cells. CD48^{-}CD150^{+} were selected (left hand panel) and the cells staining positively for Sca-1 were divided into low and high expression (right hand panel). FACs plot as in Figure 4a, shown here for reference. B) Histograms showing the MFI for each additional HSC marker (CD34 (negative), c-Kit

(positive), FLT3 (negative), and EPCR (positive)) are shown for both SLAM Sca^{lo} (upper, blue) and SLAM Sca^{hi} (lower, blue) cells. The blue populations are overlaid onto the red, the latter showing the cell distribution of each marker for the whole bone marrow. Notably, both SLAM Sca^{lo} and SLAM Sca^{hi} express all HSC markers as expected.

Figure S5, related to Figure 5; Linking molecular states with functional outcomes.

A) Sca-1 expression overlaid over the subset of sequenced cells shown in Figure 5d. The cells in the lower left-hand cluster have significantly higher Sca-1 expression and significantly higher MolO score compared to the cells in the upper right-hand cluster. Axes are in arbitrary units. B) Joint t-SNE representation of RNA-Seq data and functional data coloured according to SuMO score. Axes are in arbitrary units. C) t-SNE for 4 of the 5 patients, showing distinct clusters of mammary luminal progenitor cells defined by hierarchical clustering in the 2D t-SNE space; two of the bottom 3 clusters (5 and 6) are enriched for colony-forming cells. D) t-SNE with overlay of luminal colony-forming. Red circles indicate successful colony formation, blue circles did not form colonies. (Over-representation of colony presence in clusters 5 and 6, p=0.00001). E) Bar graph showing the colony formation across all cells and the post-enrichment colony formation (bottom clusters only) in individual patient samples (A-D). A 5th sample was not enriched using the clustering tool. F) Differential expression between enriched (5 and 6) and non-enriched clusters (1, 2, 3 and 4) of the t-SNE map for the markers used to isolate the luminal progenitors.

Table S1, related to Figure 1; Fluidigm Taqman Assays.

A summary of the TaqMan assays used for single cell gene expression analysis.

Table S2, related to Figure 3; Differentially expressed genes as identified by scRNA-Seq.

The 4533 genes identified by scRNA-Seq to be differentially regulated between individual single cells and for which the biological variability exceeded technical variability.

Table S3, related to Figure 3; Differentially expressed genes ranked based on their MolO score.

Top 500 ranked scRNA-Seq genes by the MolO score. Top negatively correlated genes (NoMO). Top positively correlated genes (MolO).

Table S4, related to Figure 5; Differentially expressed genes based on their SuMO score.

The top 500 ranked scRNA-Seq genes by the SuMO score. Top negatively correlated genes (non-SuMO). Top positively correlated genes (SuMO).

Table S1: Fluidigm assays

Gene name	Assay ID
Bptf	Mm01251151_m1
Cbfa2t3h	Mm00486780_m1
Cdkn2a	Mm00494449_m1
Csf1r	Mm01266652_m1
Dnmt3a	Mm00432881_m1
Egfl7	Mm00618004_m1
Eif2b1	Mm01199614_m1
Erg	Mm01214246_m1
Ets1	Mm01175819_m1
Ets2	Mm00468977_m1
Etv6	Mm01261325_m1
Fli-1	Mm00484409_m1
Gata1	Mm00484678_m1
Gata2	Mm00492300_m1
Gata3	Mm00484683_m1
Gfi1	Mm00515855_m1
Gfi1b	Mm00492318_m1
Hhex	Mm00433954_m1
Hoxa5	Mm00439362_m1
Hoxa9	Mm00439364_m1
Hoxb4	Mm00657964_m1
Ikzf1	Mm01187882_m1
Itga2b	Mm00439768_m1
kit	Mm00445212_m1
Ldb1	Mm00440156_m1
Lmo2	Mm01281680_m1
Lyl1	Mm01247198_m1
Mecom	Mm01289155_m1
Meis1	Mm00487659_m1
Mitf	Mm01182480_m1
Mpl	Mm00440310_m1
Myb	Mm00501741_m1
Nfe2	Mm00801891_m1
Nkx2-3	Mm01199403_m1
Notch1	Mm00435249_m1
Pbx1	Mm04207617_m1
Polr2a	Mm00839493_m1
Prdm16	Mm00712556_m1
Procr	Mm00440993_mH
Runx1	Mm01213405_m1
Spi1	Mm00488142_m1
Sh2b3	Mm00493156_m1
Smarcc1	Mm00486224_m1
Tal1	Mm01187033_m1

Tcf7	Mm00493445_m1
Tet2	Mm00524395_m1
UBC	Mm01201237_m1
Vwf	Mm00550376_m1

Supplemental Experimental Procedures

Purification of Stem and Progenitor Cells.

Suspensions of BM cells from the femurs, tibiae and iliac crest of 8–12-week-old C57BL/6 mice were isolated and depleted of red blood cells by an ammonium chloride lysis step (STEMCELL Technologies, STEMCELL)). HSCs were isolated using the following antibodies: CD45-FITC or APC-Cy7 (Clone 30-F11 Biolegend), EPCR-PE (Clone RMEPCR1560**,** STEMCELL), CD150-Pacific Blue or PE-Cy7 (Clone TC15-12F12.2, Biolegend), CD48-APC (Clone HM48-1, Biolegend), Sca-1-Pacific Blue or PE (Clone E13-161.7, Biolegend), FLT3-PE or PE-Cy5 (Clone A2F10, eBioscience), CD34-FITC (Clone RAM34, BD Biosciences), c-kit APC-Cy7 (Clone 2B8, Biolegend), and a panel of lineage markers (Hematopoietic Progenitor Enrichment Cocktail, STEMCELL) plus streptavidin-V500 (BD Biosciences). The side population (SP) cells were isolated according to the detailed protocol found at https://www.bcm.edu/research/labs/goodell/index.cfm?pmid=20017 and as previously published (Challen et al., 2012). The Hoechst dye was obtained from Sigma and used at a final concentration of 5 μg/ml. Cells were sorted using a BD Influx sorter equipped with 355 nm, 405 nm, 488 nm, 561 nm, and 640 nm lasers. For single cell gene expression assays, cells were sorted into individual wells of 96 well PCR plates using a modified plate holder for increased stability. For single cell transplantation and *in vitro* assays, cells were sorted into individual wells of a U-bottom 96 well plate. For progenitor colony forming cell assays and 10-cell transplantation assays, cells were sorted into 1.5 ml tubes containing serum free media.

Progenitor Cell Assays

500 CMPs (Lin^{-}c-kit^{+}Sca-1^{-}CD34^{+}FcγR^{low}), MEPs (Lin^{-}c-kit^{+}Sca-1^{-}CD34^{-}FcγR^{low}), and GMPs (Lin^{-}c-kit^{+}Sca-1^{-}CD34^{+}FcγR^{hi}) were isolated into 1.5ml Eppendorf tubes containing serum-free medium. Cells were then divided into a high concentration fraction (~450 cells) and a low concentration fraction (~45 cells) and placed into a semi-solid medium containing growth factors to support growth of all myeloid colony types (MC3434, STEMCELL). Both high and low concentration fractions were spread across 3 individual wells of a 6 well plate and counted after 10 and 14 days of culture. Single LMPPs (Lin^{-}c-kit^{+}Sca-1^{+}CD34^{+}FLT3^{+}) were sorted into individual wells containing OP9 cells supplemented with 100 ng/ml IL-7 and 50ng/ml FLT-3. All wells were harvested at day 28 and analyzed for the presence of B- (defined as B220+) and myeloid (Ly6g and/or Mac1) cells.

Single HSC Cultures

SLAM Sca^{hi} and SLAM Sca^{lo} HSCs were sorted and cultured in STEMSPAN medium containing 300 ng/ml SCF, 20 ng/ml IL-11, Glutamine, Penicillin, Streptomycin, β-2-mercaptoethanol and 10% FCS as described previously (Kent et al., 2008; Kent et al., 2013). After 24 hours, wells were scored for the presence of a single cell and counted each day to track the clonal growth of individual cells. For the immunophenotyping studies, clones were individually stained and assessed for the expression of Sca-1, c-Kit, and a panel of lineage markers along with 7-Aminoactinomycin D (7AAD, Invitrogen) to mark dead cells.

Clone Size Calculations and Antibody Information for *In Vitro* Cultures

When the clones began to appear, they were estimated to be small (50-5000 cells), medium (5000-20,000 cells), or large (20,000 or more cells). No clones had fewer than 50 cells. 10-

day clones were stained with biotinylated lineage marker antibodies (Haematopoietic Progenitor Enrichment Cocktail, STEMCELL), c-Kit-APC (BD) and Sca-1-Pacific Blue (Biolegend). To enumerate cells, a defined number of fluorescent beads (Trucount Control Beads, BD) were added to each well and each sample was back-calculated to the proportion of the total that were run through the cytometer. Small clones were not able to be assessed individually by flow cytometry and were pooled – the % of KSL cells was greater than 95%. Flow cytometry was performed on an LSRII Fortessa (BD) and all data were analyzed using Flowjo (Treestar, USA).

Single-Cell Gene Expression Analysis.

Single-cell gene expression analysis was performed as described previously (Moignard et al., 2013). Briefly, single cells were sorted by FACS directly into individual wells of 96-well plates containing lysis and preamplification mix. Reverse transcription and specific target amplification were performed in the same plates 24 hours after sorting. cDNA was diluted 1:5 with TE before qPCR on the BioMark HD. For the qPCR, Taqman assays (Life technologies) and cDNA samples were then loaded into a 48.48 Dynamic Array (Fluidigm), and then transferred to the BioMark HD for qPCR.

Bioinformatic Analysis of Single-Cell Gene Expression Data.

Single-cell expression data were collected using the Fluidigm Data Collection software. ΔCt values were calculated as previously described (Guo et al., 2010) by cell-wise normalization to the mean expression level of two housekeeping genes (Ubc and Polr2a). Briefly, Ct values were subtracted from the limit of detection of the BioMark (Ct 27) (Guo et al., 2010),

followed by subtraction of the mean Ct value of Ubc and Polr2a for each cell. The ΔCt value for genes that were not expressed was set to be 3.5 cycles more than the lowest Ct value per gene. All housekeepers, Cdkn2a and Egfl7 were removed from the dataset for downstream analysis. Cdkn2a was not expressed in any of the cell types and Egfl7 assay experienced technical issues. Hierarchical clustering was performed in R (www.r-project.org) using the hclust package and heatmap.2 from the gplots package using Spearman rank correlations and ward linkage.

t-SNE was performed in Matlab (Mathworks Inc., Natick, USA) using the Matlab implementation (http://homepage.tudelft.nl/19j49/t-SNE.html) with standard settings.

We identified MolO cells based on a weighting matrix defined by repopulation probabilities and the 2D t-SNE representation of the data. As cells with similar gene expression patterns are located in close proximity in the 2D t-SNE representation of the data, we aimed to identify a contiguous region in the t-SNE plot where cells within the region locally fulfil the mixture constraint derived from the weighting matrix. We therefore iteratively assessed the local neighbourhoods of all cells for molecular overlap and tested whether within each neighbourhood no progenitors were present and the fraction of each HSC subtype exceeded 80% of its respective weight. We introduced the leeway factor of 80% to allow for some sampling variation. Robustness analysis was performed additionally which assessed the leeway factors of 70 and 90 percent (Figure S2b and S2c). If both conditions were met, the cell was classified as MolO core cell and all cells within its neighbourhood were classified as MolO cells. We performed a grid search to determine the neighbourhood size maximising the number of density-reachable MolO core points (Ester, 1996).

Random forests were trained on the normalised Ct values of the set of genes which were assayed by single-cell gene expression and variable above technical noise in scRNA-Seq.

Training was performed on all cells from sorting strategy HSC1 and generalizability was quantified using 10-fold cross-validation (Figure S3c). For prediction of the sequenced cells, read counts had to be transformed to the same scale as normalised Ct values. As both sets of cells originated from the same sorting strategy we reasoned that the distributions of expression values of variable genes should be similar for the qPCR and the RNA-Seq data and performed quantile normalisation for the respective subsets of cells expressing each variable gene (Figure S3d). Training and testing of the classifier was performed in python 2.7 using the sklearn library.

Single-Cell RNA-Seq

Single cell RNA-Seq analysis was performed as described previously (Picelli et al., 2014). Briefly, single cells were sorted by FACS directly into individual wells of a 96-well plate containing lysis buffer. Reverse transcription and PCR amplification were performed in the same plates. The resulting PCR products were purified, the quality of the cDNA library verified and libraries were prepared using the Illumina Nextera XT DNA preparation kit. Pooled libraries were then run on the Illumina Hi-Seq 2500. Files were demultiplexed and reads aligned to the mouse genome (mm9) using STAR (Dobin et al., 2013). HTSeq (Anders, 2014) was run on all samples to assign mapped reads to genes, using Ensembl genes as a reference. Several QC steps were implicated, such that a single cell was required to have >500k unique counts mapping to annotated features, <10% of the counts mapped to mitochondria DNA and >4000 genes detected. Mapped reads were normalised using size factors as described in Brenencke et al (Brennecke et al., 2013). To estimate technical noise we further followed Brenencke et al (Brennecke et al., 2013) and fitted the relation between mean read counts and squared coefficient of variation using the ERCC spike-ins (Life

technologies) (Figure 3a i). Genes for which the squared coefficient of variation exceeded technical noise were considered variable. To analyse the robustness of PC1, we sub-sampled the data by repeating the PCA based on a random subset of 86 cells. We then quantified the consistency of the PCA representations by projecting the 5 outlier cells onto the new principal components, and calculated the correlation between the sub-sampled PC1 and PC1 from all cells. We repeated this procedure 500 times and the correlation coefficient was always greater than 0.98. If we omit the 5 outlier cells from the original PCA, the correlation coefficient was 0.983, giving good confidence in the clusters described.

Transplantation of Haematopoietic stem cells

10-cell transplantations were performed in CD45.1 lethally irradiated C57Bl/6 recipients along with 250,000 helper cells from the spleen of CD45.1/.2 mouse. All single cell transplantations were performed by standard intravenous tail vein injection of sublethally irradiated Ly5-congenic adult W41/W41 mice as previously described (Dykstra et al., 2007). Peripheral blood samples were collected from the tail vein of some mice at 4 weeks and all mice at 8, 16, and 24 weeks after transplantation and red blood cells lysed using ammonium chloride (Stemcell technologies, Stemcell). All samples were stained with the following antibody panel: Ly6g-Pacific Blue (Clone 1A8, Biolegend), Mac1-FITC (Clone M1/70, Biolegend), CD3e-PE (Clone 17A2, BD), B220-APC (Clone RA3-6B2, eBioscience), CD45.1-Alexa700 (Clone A20, Biolegend) and CD45.2-APC-Cy7 (Clone 104, BD)). Donor and recipient cells were distinguished by their expression of CD45.1 or CD45.2 and any double positive CD45.1 and CD45.2 events were excluded from the analysis of donor contributions to specific whole blood cell (WBC) subsets (leftover helper cells and/or doublets). Transplanted cells from which at least 1% of the WBCs and were derived at 16

and/or 24 weeks after transplantation were considered to be repopulated with long-term reconstituting cells. HSCs were further discriminated according to previously described high (alpha or beta) or low (gamma or delta) ratios of their proportional contributions to the GM, B- and T-cell subsets of the circulating WBCs assessed at 16 weeks after transplantation (Dykstra et al., 2007). Flow cytometry was performed on an LSRII Fortessa (BD) and all data were analyzed using Flowjo (Treestar, USA).

Isolation and assessment of mammary progenitors

All primary human material was derived from 5 reduction mammoplasties at Addenbrooke's Hospital, Cambridge, UK, under full informed consent and in accordance with the National Research Ethics Service, Cambridgeshire 2 Research Ethics Committee approval (08/H0308/178) as part of the Adult Breast Stem Cell Study. All tissue donors had no previous history of cancer and were premenopausal (ages 20 to 23). Mammary tissue was dissociated to single cell suspensions as previously described (Eirew et al., 2010). Single cell suspensions of human mammary cells were treated to detect the enzyme activity of aldehyde dehydrogenase (ALDH) using the Aldefluor Kit (StemCell Technologies) as per the manufacturer's instructions. The cells were incubated with the following primary antibodies: CD49f-PE/Cy7 (clone: GoH3), epithelial cell adhesion molecule (EpCAM)-PE (clone: 9C4), and CD45-APC-Cy7 (clone: HI30), CD31-APC-Cy7 (clone: WM-59) (collectively known as Lin-APC-Cy7), as well as 4',6-diamidino-2-phenylindole (DAPI) for viability. Hank's balanced salt solution supplemented with 2% FBS (Gibco) was used as the diluent for all antibody incubation and washing steps. Cells were sorted using a BD Influx. Single-stained control cells were used to perform compensation manually and gates were set in reference to fluorescence minus-one-controls. The ALDH+ gate was set in reference to control

populations incubated with the ALDH inhibitor, DEAB in addition to Aldefluor. Luminal progenitor populations were seeded single cell into 96 well plates with 1 × 10^4 irradiated NIH-3T3 feeder cells. The cultures were maintained in Human EpiCult-B (StemCell Technologies) supplemented with 5% FBS (StemCell Technologies) and 50 μg/ml gentamicin and maintained for 10 to 12 days. Colony presence was scored under a microscope.

Supplemental References

Anders, S., Pyl, P, T., and Huber, W. (2014). HTSeq - A Python framework to work with high-throughput sequencing data. bioRxiv.

Brennecke, P., Anders, S., Kim, J.K., Kolodziejczyk, A.A., Zhang, X., Proserpio, V., Baying, B., Benes, V., Teichmann, S.A., Marioni, J.C., *et al.* (2013). Accounting for technical noise in single-cell RNA-seq experiments. Nat Methods *10*, 1093-1095.

Challen, G.A., Sun, D., Jeong, M., Luo, M., Jelinek, J., Berg, J.S., Bock, C., Vasanthakumar, A., Gu, H., Xi, Y., *et al.* (2012). Dnmt3a is essential for hematopoietic stem cell differentiation. Nat Genet *44*, 23-31.

Dobin, A., Davis, C.A., Schlesinger, F., Drenkow, J., Zaleski, C., Jha, S., Batut, P., Chaisson, M., and Gingeras, T.R. (2013). STAR: ultrafast universal RNA-seq aligner. Bioinformatics *29*, 15-21.

Dykstra, B., Kent, D., Bowie, M., McCaffrey, L., Hamilton, M., Lyons, K., Lee, S.J., Brinkman, R., and Eaves, C. (2007). Long-term propagation of distinct hematopoietic differentiation programs in vivo. Cell Stem Cell *1*, 218-229.

Eirew, P., Stingl, J., and Eaves, C.J. (2010). Quantitation of human mammary epithelial stem cells with in vivo regenerative properties using a subrenal capsule xenotransplantation assay. Nat Protoc *5*, 1945-1956.

Ester, M., Kriegel, H, -P,. Sander, J, Xu, X. (1996). A density-based algorithm for discovering clusters in large spatial database with noise. Proceedings of Second International Conference on Knowledge Discovery and Data Mining, 226-231.

Guo, G., Huss, M., Tong, G.Q., Wang, C., Li Sun, L., Clarke, N.D., and Robson, P. (2010). Resolution of cell fate decisions revealed by single-cell gene expression analysis from zygote to blastocyst. Dev Cell *18*, 675-685.

Kent, D.G., Dykstra, B.J., Cheyne, J., Ma, E., and Eaves, C.J. (2008). Steel factor coordinately regulates the molecular signature and biologic function of hematopoietic stem cells. Blood *112*, 560-567.

Kent, D.G., Li, J., Tanna, H., Fink, J., Kirschner, K., Pask, D.C., Silber, Y., Hamilton, T.L., Sneade, R., Simons, B.D., *et al.* (2013). Self-renewal of single mouse hematopoietic stem cells is reduced by JAK2V617F without compromising progenitor cell expansion. PLoS Biol *11*, e1001576.

Moignard, V., Macaulay, I.C., Swiers, G., Buettner, F., Schutte, J., Calero-Nieto, F.J., Kinston, S., Joshi, A., Hannah, R., Theis, F.J., *et al.* (2013). Characterization of transcriptional networks in blood stem and progenitor cells using high-throughput single-cell gene expression analysis. Nat Cell Biol *15*, 363-372.

Picelli, S., Faridani, O.R., Bjorklund, A.K., Winberg, G., Sagasser, S., and Sandberg, R. (2014). Full-length RNA-seq from single cells using Smart-seq2. Nat Protoc *9*, 171-181.
