# Supplementary material for: Combined Single-Cell Functional and Gene Expression Analysis Resolves Heterogeneity within Stem Cell Populations
Source: Cell Stem Cell. 2015 Jun 4;16(6):712–24. doi: 10.1016/j.stem.2015.04.004 (PMC4460190; doi:10.1016/j.stem.2015.04.004)
Supplement: Document S2. Article plus Supplemental Information [file mmc5.pdf]

Cell Stem Cell

Resource

Combined Single-Cell Functional and Gene Expression Analysis Resolves Heterogeneity within Stem Cell Populations

Graphical Abstract

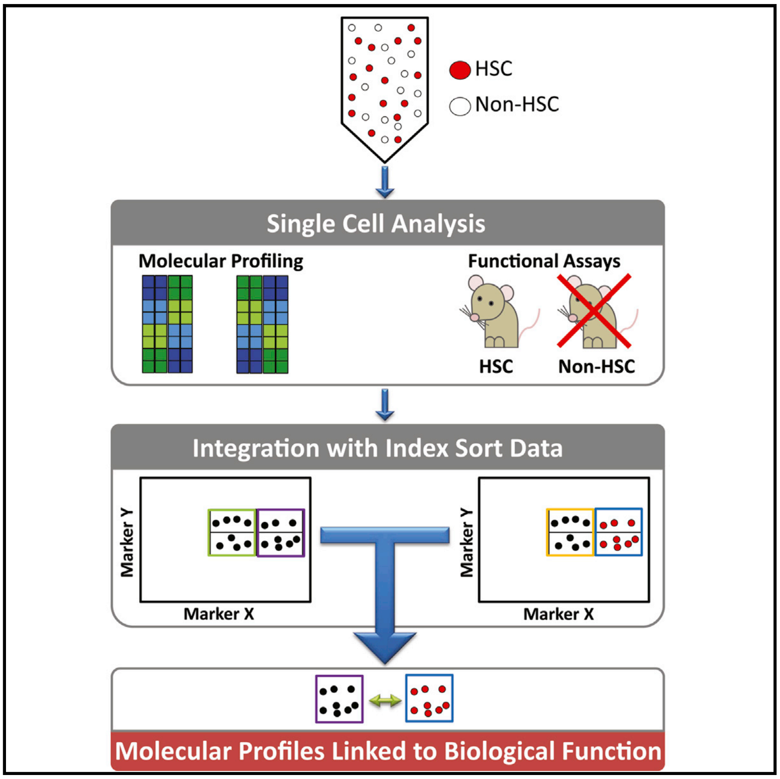

Authors

Nicola K. Wilson, David G. Kent, ..., Fabian J. Theis, Berthold Göttgens

Correspondence

bg200@cam.ac.uk

In Brief

Wilson et al. combine single-cell functional assays with flow cytometric index sorting and single-cell gene expression assays to reveal gene expression programs of HSCs with durable self-renewal potential in transplantation assays. They also demonstrate the broader applicability of this approach for linking key molecules with defined stem cell functions.

Highlights

- Comparing HSCs purified with four methods identifies key functional molecules
- Index sorting links single-cell RNA-seq with single-cell transplantation
- $EPCR^{hi}CD48^{-}CD150^{+}Sca^{hi}$ purifies HSCs with durable self-renewal
- Single-cell biology links mammalian stem cell function with markers and pathways

Accession Numbers

GSE61533

Wilson et al., 2015, Cell Stem Cell *16*, 712–724
June 4, 2015 ©2015 The Authors
http://dx.doi.org/10.1016/j.stem.2015.04.004

Cell Stem Cell
Resource

Combined Single-Cell Functional and Gene Expression Analysis Resolves Heterogeneity within Stem Cell Populations

Nicola K. Wilson,[1,9] David G. Kent,[1,9] Florian Buettner,[2,9] Mona Shehata,[7] Iain C. Macaulay,[3] Fernando J. Calero-Nieto,[1] Manuel Sánchez Castillo,[1] Caroline A. Oedekoven,[1] Evangelia Diamanti,[1] Reiner Schulte,[4] Chris P. Ponting,[3,5] Thierry Voet,[3,6] Carlos Caldas,[7] John Stingl,[7] Anthony R. Green,[1] Fabian J. Theis,[2,8] and Berthold Göttgens[1,*]
[1]Department of Haematology, Wellcome Trust and MRC Cambridge Stem Cell Institute and Cambridge Institute for Medical Research, Cambridge University, Cambridge CB2 0XY, UK
[2]Institute of Computational Biology, Helmholtz Zentrum München, Ingolstädter Landstraße 1, 85764 Neuherberg, Germany
[3]Single Cell Genomics Centre, Wellcome Trust Sanger Institute, Hinxton, Cambridge CB10 1SA, UK
[4]Head of Flow Cytometry, Cambridge Institute for Medical Research, Cambridge University, Cambridge CB2 0XY, UK
[5]MRC Computational Genomics Analysis and Training Programme, MRC Functional Genomics Unit, Department of Physiology, Anatomy and Genetics, University of Oxford, Oxford OX1 3PT, UK
[6]Laboratory of Reproductive Genomics, Department of Human Genetics, KU Leuven, 3000 Leuven, Belgium
[7]Department of Oncology and Cancer Research UK Cambridge Institute, University of Cambridge, Li Ka Shing Centre, Cambridge CB2 0RE, UK
[8]Department of Mathematics, Technische Universität München, Boltzmannstraße 3, 85748 Garching, Germany
[9]Co-first author
*Correspondence: bg200@cam.ac.uk
http://dx.doi.org/10.1016/j.stem.2015.04.004
This is an open access article under the CC BY license (http://creativecommons.org/licenses/by/4.0/).

SUMMARY

Heterogeneity within the self-renewal durability of adult hematopoietic stem cells (HSCs) challenges our understanding of the molecular framework underlying HSC function. Gene expression studies have been hampered by the presence of multiple HSC subtypes and contaminating non-HSCs in bulk HSC populations. To gain deeper insight into the gene expression program of murine HSCs, we combined single-cell functional assays with flow cytometric index sorting and single-cell gene expression assays. Through bioinformatic integration of these datasets, we designed an unbiased sorting strategy that separates non-HSCs away from HSCs, and single-cell transplantation experiments using the enriched population were combined with RNA-seq data to identify key molecules that associate with long-term durable self-renewal, producing a single-cell molecular dataset that is linked to functional stem cell activity. Finally, we demonstrated the broader applicability of this approach for linking key molecules with defined cellular functions in another stem cell system.

INTRODUCTION

Hematopoiesis is one of the best described models of adult stem cell biology due to the accessibility of tissue and the ability to isolate and functionally characterize multiple stages of a clearly defined hierarchy of differentiation (Bryder et al., 2006; Ema et al., 2014). HSCs can divide symmetrically, producing two HSCs or two progenitor cells, or asymmetrically, giving rise to an HSC and a progenitor cell. On a population level, these fate choices must be tightly regulated to maintain the HSC pool size throughout life while still supplying the required numbers and types of mature blood cells needed by the organism. Single-cell and serial transplantation studies have revealed significant heterogeneity in both the mature cell production and self-renewal durability of individual HSCs (Beerman et al., 2010; Dykstra et al., 2007; Goodell et al., 1996; Morita et al., 2010). This functional heterogeneity is thought to be controlled via cell intrinsic and extrinsic mechanisms (Copley and Eaves, 2013; Wilkinson and Göttgens, 2013) and is thought to play a role in disease evolution (Prick et al., 2014).

Advances in multiparameter flow cytometry have permitted isolation of HSCs for single-cell functional assays of cellular fate choice (Dykstra et al., 2007; Kent et al., 2008; Naik et al., 2013; Rieger et al., 2009). Because of the retrospective nature of these assays, individual cells shown to possess HSC properties are no longer available for molecular analyses. A long-standing goal in the field has been the identification of phenotypically and functionally pure HSCs, both in terms of cell surface marker expression and regenerative capacity upon transplantation. While this has led to the identification of dozens of markers that enrich for HSC populations containing long-term HSCs (LT-HSCs), it is unclear which cells are HSCs and which are contaminating cells within any given HSC-enriched population.

To address the issue of molecular and functional heterogeneity in HSCs, we took an integrated single-cell approach. Using four commonly used HSC purification strategies, we performed single-cell gene expression in combination with flow cytometric

index sorting. We report the molecular signature for these four HSC populations and present the integration of these data with indexed flow cytometry data and single-cell RNA-seq (scRNA-seq) alongside in vitro and in vivo functional assays. Subsequent integration of these datasets permitted design of an unbiased sorting strategy that separates non-HSCs away from HSCs. Single-cell transplantation experiments using the enriched population were then undertaken and combined with the RNA-seq data to identify key molecules that associate with long-term durable self-renewal to produce a single-cell molecular dataset that is linked to functional stem cell activity.

RESULTS

Single-Cell Gene Expression Analysis Reveals an Overlapping Molecular Signature for Four Heterogeneous HSC Populations

The most refined HSC purification strategies can now isolate HSCs at 40%–50% purity as validated by single-cell transplantation experiments (Beerman et al., 2010; Challen et al., 2010; Kent et al., 2009; Kiel et al., 2005; Morita et al., 2010). While each strategy identifies some fraction of functional HSCs, not all cells are able to repopulate an irradiated mouse. To identify commonalities between populations, we selected four widely used HSC isolation strategies (Adolfsson et al., 2001; Kent et al., 2009; Kiel et al., 2007; Weksberg et al., 2008) in addition to a finite self-renewal HSC (FSR-HSC) fraction (Kent et al., 2009) and four defined progenitor populations, lymphoid-primed multipotent progenitors (LMPPs) (Adolfsson et al., 2005), common myeloid progenitors (CMPs), megakaryocyte-erythroid progenitors (MEPs), and granulocyte-monocyte progenitors (GMPs) (Akashi et al., 2000) (Figures 1A and S1A). Progenitor populations were included to further resolve HSC fractions in terms of self-renewal and multilineage capacity. We isolated over 1,800 cells for single-cell gene expression analysis (n = 210 per population) and validated each population by functional assays, as outlined below. For CMP, GMP, and MEPs, 500 cells were isolated and placed into methylcellulose cultures, while single LMPPs were sorted onto OP9 feeder cells in 96-well plates, as described previously (Månsson et al., 2007) (Figure S1B). Clonal assays were performed for all populations and functional readouts were 65% CMPs, 60% GMPs, 38% MEPs, and 45% LMPPs, in line with previous publications. For each HSC population, 50 cells were isolated and transplanted into five lethally irradiated recipients (e.g., an average of ten cells per mouse). All four strategies contained HSCs at a frequency of one in ten or greater, as estimated by the extreme limiting dilution tool (http://bioinf.wehi.edu.au/software/elda/), with two populations repopulating all mice transplanted (Figures S1C and S1D; Kent et al., 2009). Cells for single-cell expression and functional assays were isolated on the same day from the same mouse bone marrow suspension.

Single-cell gene expression analysis of 48 genes was performed in all 1,800 cells. Our gene set included 33 transcription factors important for HSCs and hematopoiesis (Wilkinson and Göttgens, 2013), 12 additional genes implicated in HSC biology, and 3 housekeeping genes (Figure 1B; Table S1). Unsupervised hierarchical clustering revealed that the HSCs and progenitors form two distinct clusters (I and II respectively, Figure 1C). Within the progenitor cluster (II), cells were divided into five subclusters, which separate the LMPPs (IIa) and MEPs (IIc). The GMPs are divided in two locations, with one subset of cells (IIb) clustering next to the LMPPs (IIa) and the second subset (IId) between the MEPs (IIc) and a number of HSC-sorted cells (IIe). CMPs tended to be interspersed within the GMP clusters (IIb and IId). Clusters IIa and IIb had higher expression of Pu.1 (Spi1), whereas IIc, IId, and IIe have higher expression of the more erythrocyte/megakaryocyte TFs Scl/Tal1, Gfi1b, Gata1, and Gata2.

Unsupervised clustering revealed a distinct group of cells (cluster IIe) sorted using HSC gating strategies yet clustering with progenitor cells. These cells express high levels of vWF, as well as several myeloid lineage-associated genes (Gfi1b, Itga2b, Pbx1, and Mpl), potentially suggesting a bias toward the megakaryocytic lineage, as recently described (Sanjuan-Pla et al., 2013; Yamamoto et al., 2013). Of note, these cells did not cluster with any specific progenitor or HSC cluster and are only present in sorting strategies HSC1 and HSC3, which suggests that they could represent a separate entity. The HSC cluster (I) contained the vast majority of phenotypically defined HSCs (86%) and only 4% of various progenitor cells. Generally, the HSC populations overlap with each other, but individual patterns can be observed. For example, cluster Ib is primarily made up of HSC5, the HSCs with finite self-renewal, and does not express vWF as previously shown (Kent et al., 2009).

Multidimensional Analysis Can Further Resolve Cell Populations

To further analyze the multidimensional gene expression data from the 43 genes (excluding housekeepers, Cdkn2a and Egfl7, see Experimental Procedures) we performed t-distributed stochastic neighbor embedding analysis (t-SNE) (van der Maaten and Hinton, 2008), which has previously been reported as a superior method for the definition of subpopulations by cytometry time of flight (CyTOF) mass spectrometry (Amir et al., 2013). In contrast to standard linear methods such as principal component analysis (PCA), t-SNE can capture nonlinear relationships in the data. Each point on the t-SNE map represents an individual cell, and each cell is colored according to the sorting strategy described in Figure 1. The t-SNE analysis reiterates separation of the cell populations seen in the hierarchical clustering, but the scatterplot presents a clearer distribution of the populations in relation to one another (Figures 2A and S2A). The t-SNE analysis largely recapitulates the population dendrogram from Figure 1C, except that the CMPs are divided into three clusters; the majority of CMPs is distributed between the two progenitor subgroups, whereas a minority falls into a third fraction that groups preferentially with a small subset of the HSC1 population. The HSC populations are divided into five subsets of cells. The majority of three of the HSC populations are separately partitioned within the t-SNE map (HSC4 [SP^{KSL} $CD150^+$], HSC2 [E-SLAM], and HSC5 $CD45^+EPCR^+CD48^-CD150^-$). Interestingly, a cluster of cells falling in between progenitors and HSCs is comprised of HSC1 ($Lin^-c\text{-}Kit^+Sca\text{-}1^+(KSL)CD34^-Flt3^-CD48^-CD150^+$), HSC3 (KSL$CD34^-Flt3^-$), GMPs, and CMPs. There is 89% overlap with this group of cells and the cells from cluster IIe of the hierarchical clustering; 48% were sorted as progenitors, and 41% were sorted using HSC sorting parameters (Figure 1C). The remaining HSCs cluster together in the t-SNE map. These "overlapping" HSCs therefore share a common gene expression

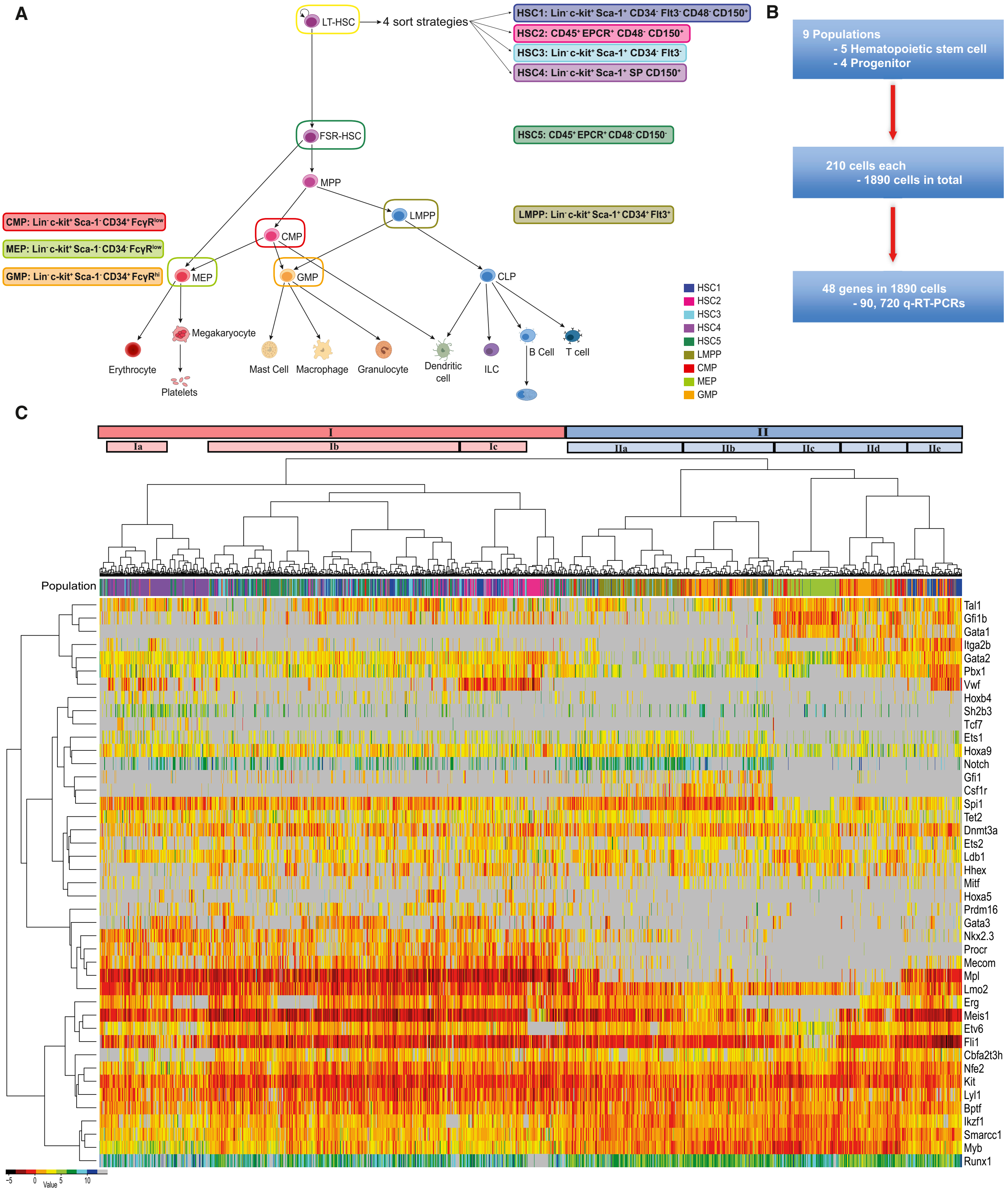

Figure 1. Single-Cell Expression Analysis Reveals an Overlapping Molecular Signature for Four Heterogeneous HSC Populations

(A) Schematic of the hematopoietic tree. The cell types highlighted are populations that will be further investigated within this study; the colors and names remain constant throughout the text. The individual sorting strategies are also highlighted next to the appropriate cell population. HSC1 (dark blue, Lin$^-$c-kit$^+$Sca-1$^+$ CD34$^-$Flt3$^-$CD48$^-$CD150$^+$), HSC2 (pink, Lin$^-$CD45$^+$EPCR$^+$CD48$^-$CD150$^+$), HSC3 (cyan, Lin$^-$c-kit$^+$Sca-1$^+$CD34$^-$Flt3$^-$), HSC4 (orchid, Lin$^-$c-kit$^+$Sca-1$^+$SP

(legend continued on next page)

profile for the 43 genes and may represent, at least partially, the subset of true HSCs present in each sorted population.

To identify common functional HSCs from the heterogeneous mix of the five populations, we developed a bioinformatic approach, based on the reasoning that a functionally homogeneous HSC subpopulation should consist of a mixture of cells from all sorting strategies, with mixture weights corresponding to the respective published probability of durable self-renewal (Figure 2B). Using these probabilities together with the 2D t-SNE representation of the cells, we identified a subpopulation of HSCs that first consists of cells that are similar in terms of their gene expression and second consists of a mixture of all HSC populations weighted by their repopulation probability (Figure 2C). By iteratively assessing the local neighborhoods of all points (see Experimental Procedures), our approach located a region within the t-SNE map that contains a defined subpopulation of HSCs (cells highlighted in red, Figure 2D). We refer to this HSC subpopulation with homogenous gene expression as the molecular overlapping population (MolO) and the HSCs outside of the identified neighborhood as cells with no molecular overlap (NoMO).

Comparing MolO and NoMO populations, we identified 28 of 43 genes, which were differentially expressed (Figure 2D). We also investigated which cell surface markers could distinguish the MolO from the NoMO population by taking advantage of index sorting (Osborne, 2011), which allows for the exact flow cytometric phenotype and location of each single cell sorted to be reviewed post-sort. The index sort data revealed that the MolO cells were enriched for higher than average CD150 and Sca-1 surface marker expression and lower than average CD48 expression (Figures 2D, S2D, and S2E). Together these data show that subsets from different phenotypically defined HSC populations share common molecular features and can be retrospectively assigned to a common cell surface phenotype.

Genome-wide Expression Data of 92 Single HSCs Reveal a MolO HSC Gene Signature

Single-cell gene expression analysis provided a precise snapshot of the dynamic gene expression levels within a heterogeneous population, but it only permits a select number of genes to be analyzed. To provide an unbiased genome-wide approach and increase the probability of gene discovery, we performed scRNA-seq (Picelli et al., 2014) for 96 single cells of the HSC1 population. Following reverse transcription, library preparation, and next generation sequencing, 92 cells passed stringent quality control measures (see Experimental Procedures) and yielded an average of 2 million uniquely mappable paired-end reads per single cell. To identify genes which were differentially regulated between individual cells, we took advantage of a recent quantitative statistical method (Brennecke et al., 2013) and determined genes for which the biological variability exceeded technical variability (Figures 3A and 3Ai). This resulted in a set of 4533 genes (Table S2). Many of the genes analyzed by the multiplexed qPCR were identified as being heterogeneously expressed in the scRNA-seq dataset (Bptf, Dnmt3a, Ets2, Fli1, Gata1, Gata3, Gfi1, Gfi1b, Hhex, Itga2b, Lyl1, Myb, Notch1, Pbx1, Procr, Spi1, and vWF) (Table S2). Next, PCA was performed based on the variable genes to visualize the distribution of the individual HSC1 cells based solely on their global gene expression profiles (Figure 3Aii). Substantial heterogeneity can be seen within the population, with principal component 1 separating the HSCs, visualized by a large number of cells shifted to the left of the PCA plot. The HSCs, which are located toward the left-hand side of the plot, are then further separated by principal component 2. The genes that influence the principal components can be seen in the loading plot (Figures 3Aiii and S3A). Genes important for component 1 include Ly6a (Sca-1), Procr (EPCR), and Pqlc3, whereas component 2 is influenced by Acap1, Cdkn1c, Clu, Ctla2a, Ctla2b, Ctnna1, Glipr 1, Muc13, Rgs1, Sultlal, and vWF.

We next used a random forest classifier (Breiman, 2001) to predict which of the 92 HSC1 single-cell RNA-seq profiles have a molecular signature similar to the intersecting MolO subpopulation identified in Figure 2C (Figure 3B). The genes with the greatest influence upon the classifier were Itga2b, vWF, Procr, Ets2, and Gata1 (Figure S3B). All HSC1 scRNA-seq cells were given a MolO score, which denotes at which confidence level the classifier can accurately determine that the individual cell is in fact a MolO cell (Figure 3C). The cells with the highest MolO score are located in the top left-hand side of the PCA plot with strong correlation between PC1 and MolO scores ($p = 4.5e-7$) and, separation of cells on the PCA plot is driven by genes such as Ly6a, Procr, Slamf1, and vWF (Figure 3Aiii). We next ranked on a transcriptome-wide level all 4,533 differentially expressed genes based on their MolO score (Table S3). Following correction for multiple testing using the Benjamini-Hochberg method, a total of 75 genes were found to be significantly negatively correlated to the MolO score and consequently more highly expressed in the NoMO population (Figure 3D). Gene ontology (GO) analysis identified the cell cycle to be an overrepresented functional category (colored in red). Twenty-nine genes showed significant positive correlation to the MolO score and were therefore overexpressed in the MolO population. Three of these genes (Cdkn1c, Ptpn14, and Ifitm1) are associated with negative regulation of cell proliferation (colored yellow). Together these data show that at least two distinct molecular clusters are present in the HSC1 population, one primed for proliferation and the other enriched for genes that would negatively regulate proliferation.

Single-Cell Assays Affirm High Proliferation and Differentiation of NoMO HSCs

We had previously seen that the MolO cells had higher than average CD150 and Sca-1 expression and lower than average

$CD150^{+}$), HSC5 (seagreen, $Lin^{-}CD45^{+}EPCR^{+}CD48^{-}CD150^{-}$), LMPP (yellow, Lin^{-}c-kit^{+}Sca-$1^{+}CD34^{+}Flt3^{+}$), CMP (red, Lin^{-}c-kit^{+}Sca-$1^{-}CD34^{+}Fc\gamma R^{low}$), MEP (yellow-green, Lin^{-}c-kit^{+}Sca-$1^{-}CD34^{-}Fc\gamma R^{lo}$) and GMP (orange, Lin^{-}c-kit^{+}Sca-$1^{-}CD34^{+}Fc\gamma R^{hi}$).

(B) Flow diagram of single-cell qRT-PCR.

(C) Unsupervised hierarchical clustering of gene expression for all investigated cell populations. Colored bar (population) above heat map indicates the cell population (colors are the same as in A). Intensity of heat map is based on the ΔCt, black is highest expressed—dark blue is lowest, and gray is not detected. The distances of the population dendrogram are not proportional to the dissimilarity.

See also Figure S1 and Table S1.

A

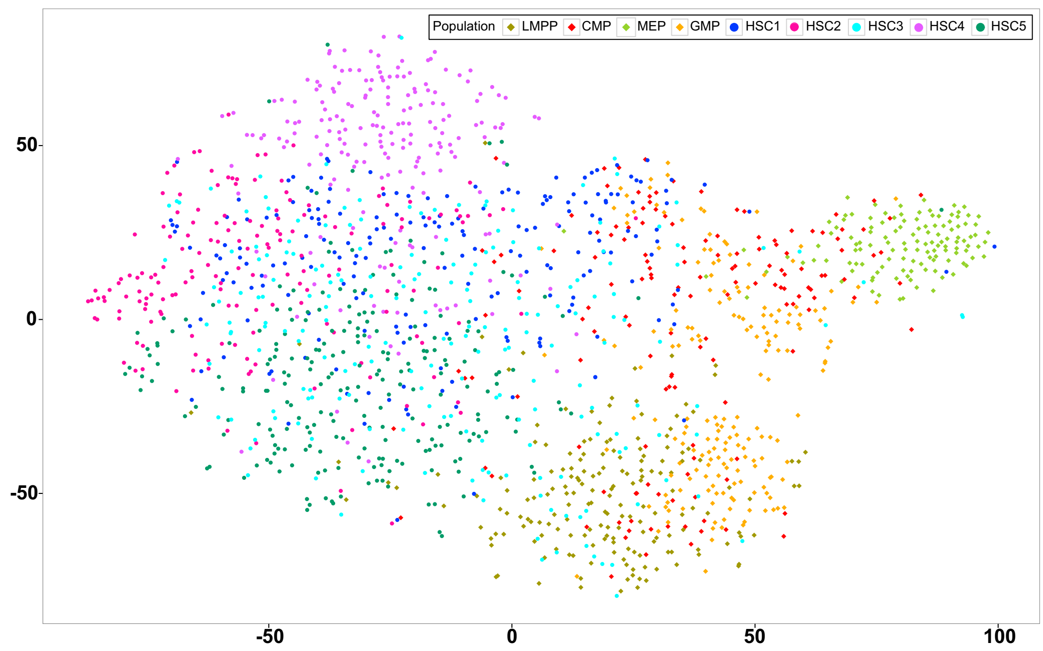

B

Population	Long term repopulating cell (%)	Durable (%)	Weighting
HSC1	50	50	0.352
HSC2	55	40	0.281
HSC3	35	25	0.176
HSC4	30	20	0.141
HSC5	40	7	0.049

C

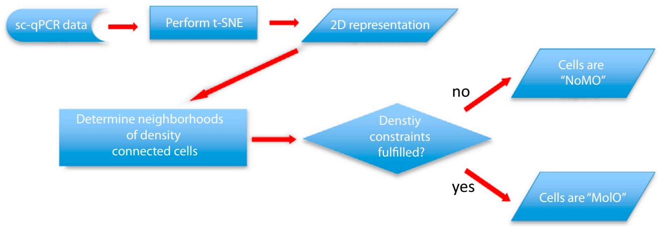

D

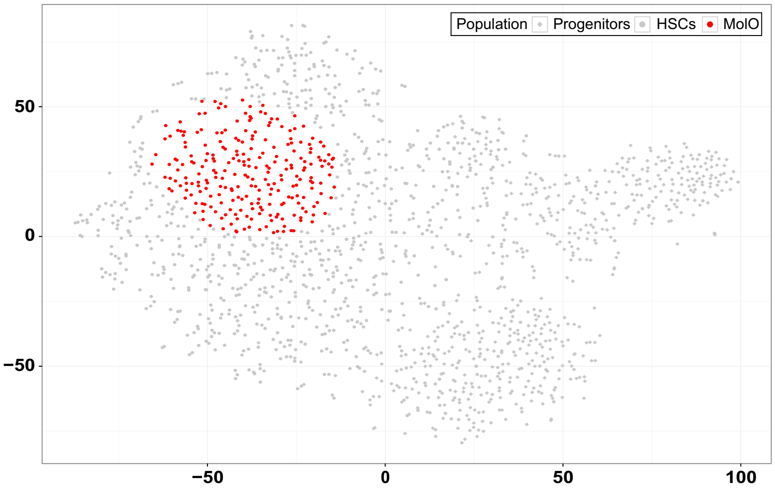

MolO vs. NoMO				
Q-RT-PCR				FACS
Cbfa2t3	Gata2	Kit	Notch	CD150
Erg	Gata3	Lmo2	Pbx1	Sca-1
Ets1	Gfi1	Lyl1	Prdm16	CD48
Ets2	Gfi1b	Mecom	Procr	
Etv6	Hoxb4	Meis1	Tal1	
Fli1	Ikzf1	Mpl	Tet2	
Gata1	Itga2b	Nfe2	vwf	

Figure 2. Multidimensional Analysis Can Further Resolve Cell Populations

(A) t-SNE plot of all cells calculated from the 43 genes analyzed by Fluidigm. All HSCs are circles and all progenitors are diamonds. Axes are in arbitrary units.

(B) Table of the published repopulation data used for the weighting program and schematic of the computational weighting program.

(C) Schematic showing the definition of MolO cells.

(D) t-SNE plot as in (A) with the MolO HSCs identified by the computational weighting highlighted in red. Axes are in arbitrary units. Table showing differentially regulated genes between MolO and NoMO populations. Red, genes upregulated in MolO population; blue, genes downregulated in MolO population.

See also Figure S2.

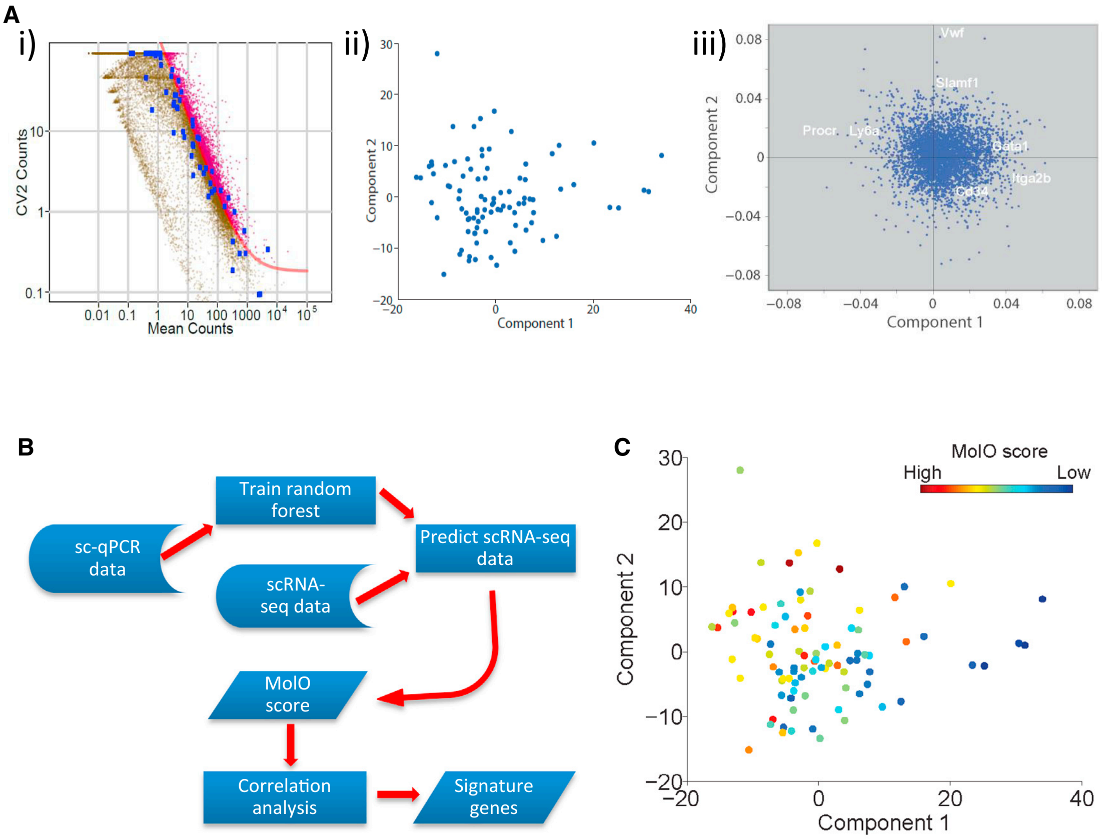

D

NoMO								MolO		
2810417H13 Rik	Ccne2	Dtnbp1	Ints2	Nasp	Rasa2	Sh2d5	Trip13	Cd82	Limd2	Ptpn14
Aqp1	Cdca8	Fam64a	Itga2b	Ncapg	Rasal3	Shcbp1	Ube2c	Cdkn1c	Ltb	Ramp2
Aqp9	Cdk6	Gata1	Kif2c	Ndrg1	Rnf168	Ska1	Uhrf1	Cldn10	Ly6a	Smtnl1
Arhgap27	Cenpf	Gja1	Kif4	Nek2	RP23-81C12.1	Slc22a3	Vps18	Ctsf	Mettl7a1	Sox18
Arhgap5	Cenph	Glipr1	Knstrn	Nkg7	Rrm1	Spag5	Yme1l1	Fads3	Mllt3	Sqrdl
Asf1b	Cenpi	Gp1bb	Mdm1	Nuf2	Sdf2l1	Spc24		Fgfr3	Neil2	Sult1a1
Aspm	Ckap2	Gp5	Mfsd2b	Pa2g4	Serpinb1a	Spred1		Gimap1	Neo1	Trim47
Birc5	Ckap2l	Gpd2	Mis18bp1	Pbk	Sgol1	Syk		Gimap6	Pde1b	Ubl3
Cacybp	Csgalnact1	Hmmr	Mki67	Pf4	Sgpp1	Top2a		Gstm1	Pdzk1ip1	Vwf
Ccna2	Dlgap5	Hsp90aa1	Muc13	Plk1	Sh2d3c	Tor1b		Ifitm1	Procr	

Cell cycle Negative regulation of cell proliferation

Figure 3. Genome-wide Expression Pattern of 92 Single HSCs Reveals a Gene Signature for the MolO Population

(A) RNA-seq analysis. (i) Identification of variable genes across all 92 cells. The genes highlighted in magenta have a coefficient of variation exceeding technical noise. The blue dots represent the distribution of the internal control ERCC spike-ins. (ii) PCA plot for the 92 cells analyzed by RNA-seq, showing the first and second components for all genes which were identified to be variably expressed. (iii) Principal component loading plot of scRNA-seq, indicating which genes also assayed by Fluidigm analysis and/or flow cytometry contribute to the separation of the cells along each component.

(B) Schematic showing the principle of the classifier to determine the MolO HSCs from the scRNA-seq dataset.

(C) PCA plot showing MolO score.

(D) Table of signature genes differentially expressed in either NoMO or MolO cells following correction for multiple testing at a false discovery rate (FDR) of 0.1. Coloring relates to the GO term associated with the gene: red, cell cycle; yellow, negative regulation of cell proliferation.

See also Figure S3 and Tables S2 and S3.

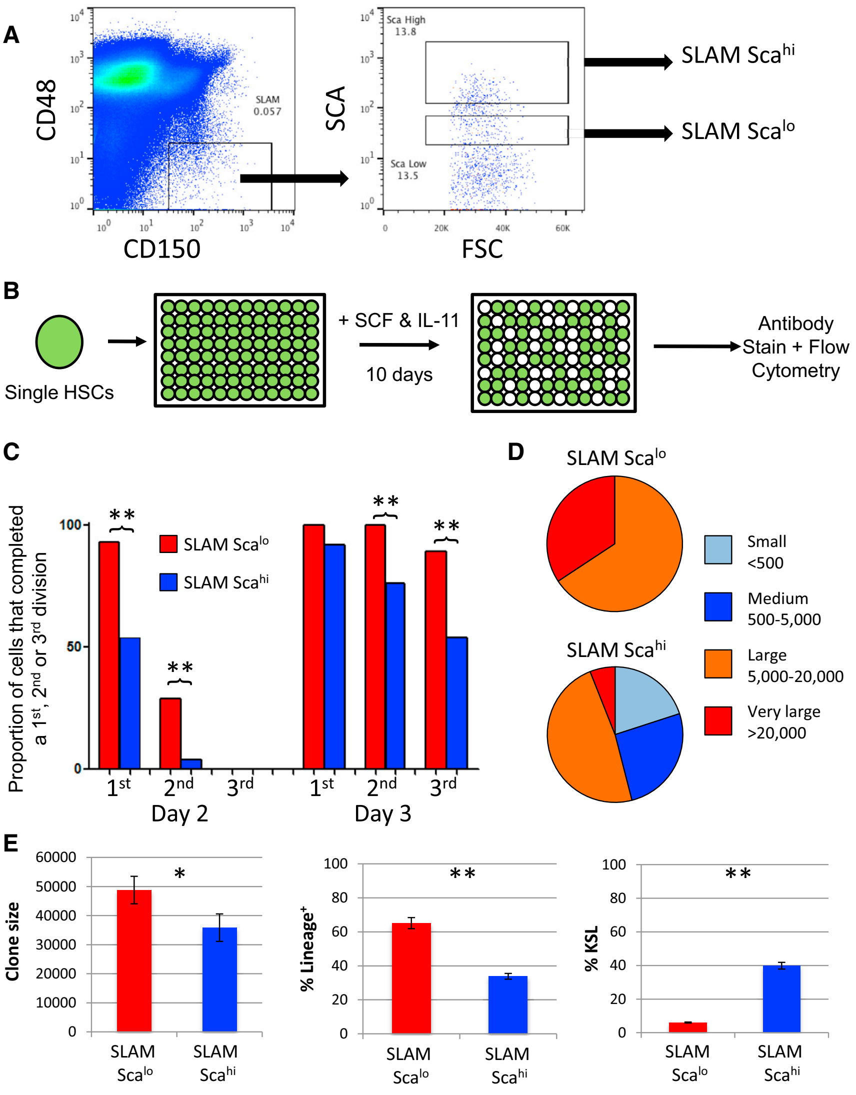

Figure 4. SLAM Sca^{lo} Cells Make Large Differentiated Clones Compared with SLAM Sca^{hi} Cells

(A) The most discriminating sequence of surface markers resulted in the sorting strategy shown on the right, which first selects CD48^{-}CD150^{+} cells and then partitions the Sca positive cell fraction into high (SLAM Sca^{hi}) and low (SLAM Sca^{lo}) levels. The negative Sca-1 population was set at less than 10^1, meaning all cells were Sca1^{+}.

(B) Schematic for single cell in vitro study where single HSCs were cultured in SCF and IL-11 for 10 days and analyzed by flow cytometry.

(C) The bar graph shows the cumulative number of cells that reached the first, second, and third division on each of the first four days of culture. First division was determined by the presence of two cells, second by three or more cells, and third by five or more cells. Notably, the SLAM Sca^{hi} population entered division significantly later and also had fewer second and third division clones on days 2–4.

(D) The pie charts depict the ratio of small (<500), medium (500–5,000), large (5,000–20,000), and very large (>20,000) clones formed from single SLAM Sca^{lo} (upper chart) and SLAM Sca^{hi} (lower chart). All clones formed by single SLAM Sca^{lo} cells were large or very large.

(E) Clones were assessed by flow cytometry, and accurate clone sizes were determined using a standard number of fluorescent beads in each well and then back calculated to get total clone size. The clone size (left), percentage of lineage marker expression (middle), and percentage of KSL cells (right) are shown. Notably, SLAM Sca^{hi} clones are smaller and less differentiated. Error bars represent data ± SEM.

See also Figure S4. *$p < 0.05$, **$p < 0.01$.

CD48 expression. Based on this finding, we designed a sorting strategy to distinguish between MolO and NoMO cells. We first gated on CD48^{-}CD150^{+} cells and then separated this population based on high or low Sca-1 expression (Figure 4A). Importantly, all cells, including the "SLAM Sca^{lo}" cells, were clearly Sca-1 positive, and SLAM Sca^{lo} cells still expressed the other markers typical of HSCs (Figure S4). This analysis provided a sorting strategy specifically designed to enrich for MolO cells in the most simple and discriminatory way possible. In order to validate both the molecular classification and the surface marker phenotype, we performed single-cell assays on the newly defined MolO cell-sorting strategy. We first cultured individual SLAM Sca^{lo} and SLAM Sca^{hi} cells in culture conditions previously used to determine the proliferation and differentiation characteristics of single HSCs (Dykstra et al., 2007; Kent et al., 2008, 2013) (Figure 4B). SLAM Sca^{lo} cells entered the cell cycle more rapidly than SLAM Sca^{hi} cells (Figure 4C), and when 10-day clones were scored for size, the only small (<500 cells) and medium (500–5,000 cells) clones observed were from the SLAM Sca^{hi} fraction (Figure 4D). All medium, large (5,000–20,000 cells), and very large clones (>20,000 cells) were next assessed individually by flow cytometry. Those originating from a SLAM Sca^{lo} cell expressed more mature lineage markers and contained fewer KSL cells compared with SLAM Sca^{hi} cells (Figure 4E). Together these data show that SLAM Sca^{hi} cells have low proliferation and low differentiation characteristics compared with SLAM Sca^{lo} cells, consistent with the cellular behavior predicted by the MolO gene expression profile.

To confirm that in vitro culture of sorted MolO HSCs correlated with in vivo HSC properties, we undertook transplantation experiments of SLAM Sca^{lo} and SLAM Sca^{hi} cells. Whereas all mice receiving 10 SLAM Sca^{hi} cells had robust multilineage donor repopulation at 16 weeks, those receiving 10 SLAM Sca^{lo} cells had lower chimerism ($p < 0.05$, t test; Figure 5A) with four of five having fewer than 1% myeloid cells, strongly predictive of a low success in secondary transplantation experiments (Figure 5B). We investigated whether this was due to differences in cell-cycle status or homing, but no differences were observed (data not shown). We next transplanted 29 mice with single SLAM Sca^{hi} cells to quantify HSC frequency. Fifteen of 29 mice receiving a single SLAM Sca^{hi} cell gave rise to long-term multilineage reconstitution (Figure 5C). Two of these HSCs would be classified as a

gamma subtype HSC, meaning that they are lymphoid biased and unlikely to possess durable self-renewal activity (i.e., not able to reconstitute in a secondary transplantation). Interestingly, Grinenko et al. (2014) recently described c-Kit levels as a robust marker of HSCs, with intermediate levels of c-Kit associating with durable self-renewal potential. In agreement with this study, the SLAM Sca^{hi} cells show a modest but consistent reduction in c-Kit mean fluorescence intensity (MFI) values compared with SLAM Sca^{lo} cells (data not shown).

A Refined HSC Molecular Profile Based on Single-Cell Function

Since all of the SLAM Sca^{hi} cells used in the single-cell transplantation experiments were also index sorted with readings for 11 flow cytometry parameters recorded for every single cell, we used the index data to link HSC functional capacity (i.e., positive transplantation readout) with the RNA-seq data in Figure 3. Importantly, relative intensities for the same 11 flow cytometry parameters (FSC, SSC, 7AAD, Sca-1, Lin, CD34, EPCR, FLT3, CD48, CD150, and c-Kit) were obtained for both the single cells used in the RNA-seq and transplantation experiments. This allowed the definition of a population of cells with surface marker overlap (SuMO cells), containing both cells for which functional information is available as well as cells for which transcriptional information is available. We performed t-SNE analysis on the 92 single cells analyzed by scRNA-seq together with the 29 cells assayed by single-cell transplantation experiments (Figure 5D). The resulting 2D representation of the cells based on their surface marker expression only resulted in two major clusters of cells (top right-hand and lower left-hand portions of the plot). Single cells from the RNA-seq dataset with high MolO scores were significantly enriched ($p = 0.0003$, Wilcoxon rank sum test) in the lower left-hand portion of the plot. Moreover, the majority (12 of 15) of the functional HSCs were also found in this lower left-hand portion of the plot. This region was also enriched for Sca-1^{hi} cells ($p < 0.0001$, Wilcoxon rank sum test), with Sca-1 being the surface marker best able to discriminate between the two regions (Figure S5A). The SuMO score (capturing the overall phenotype) was significantly correlated ($p < 0.0001$, Spearman rank correlation) with the MolO score, representing the probability of reading out as functional HSCs based on gene expression results.

One of the three repopulating HSCs not associated with a high MolO score was a gamma-HSC (finite self-renewal), and another was a balanced beta-HSC with just 1% chimerism, both representing the lower end of qualitative HSC activity. Nine of the 14 non-repopulating cells were associated with a low MolO score and located in the upper portion of the t-SNE plot, suggesting that these cells may differ in terms of their cell surface marker expression. Notably, 5 of the 14 cells determined to be non-repopulating HSCs clustered with those cells with high MolO scores, and one of these showed characteristics of an alpha-HSC described by Dykstra et al. (2007) with 0.4% chimerism dominated by elements of the myeloid system (but did not meet the 1% criteria we set for HSC repopulation). The remaining four cells showed no traces of donor cells and possibly reflect the limitations of the single-cell transplantation assay itself where a cell may remain in the syringe or die within the first few hours of transplantation. Together, these data strongly link a specific subset of scRNA-seq libraries with functional transplantation outcomes and reinforce the strength of the MolO scoring metric.

To further resolve the functional HSC population using the single-cell RNA-seq data, we applied single-cell latent variable model (scLVM), a recently proposed framework for the computational dissection of gene expression heterogeneity (Buettner et al., 2015). Briefly, we used known gene sets to estimate latent factors representing hidden sources of variation and then decomposed the observed gene expression variability on a gene-by-gene basis. We separated the variation into technical noise (estimated using External RNA Controls Consortium spike-ins), variations in cell size (from the flow cytometry index data), differentiation related processes, homing, apoptosis, and interaction between differentiation and apoptosis. The largest contributor (111 genes) was the interaction factor between differentiation and apoptosis, supporting the link between differentiation and proliferation identified in the NoMO cell population. We then assessed the correlation between the hidden factors representing apoptosis and differentiation with surface markers and found that apoptosis had a weak but significant correlation with Sca-1 ($p = 0.001$), while differentiation was significantly (negatively) correlated with EPCR ($p < 0.0001$; Figure 5E). Based on this analysis, we refined our single-cell sorting gates and undertook an additional 39 single-cell transplantations using EPCR^{hi} in addition to SLAM Sca^{hi}; 67% (26 of 39) single-cell transplantations gave rise to long-term multilineage clones at 16–24 weeks, representing a near pure population of HSCs (Figure 5F).

We next derived a SuMO score from the 2D t-SNE representation of the high-dimensional surface marker expression data by fitting a linear model through the cells in 2D (Figure S5B). When compared with the scRNA-seq libraries that associate with a greater number of non-repopulating HSCs, a specific gene signature for the SuMO cells could be identified by performing a correlation analysis and assessing which genes were significantly correlated with the SuMO score. We again ranked all differentially expressed genes based on their SuMO score (Table S4) and list those genes that are significantly associated with the SuMO score (which in turn is associated with repopulating HSCs) in Figure 5G. There is a high degree of overlap between the gene lists for the MolO/NoMO and SuMO/non-SuMO cells. Importantly, the MolO score is based solely on the molecular profile, and the SuMO population is generated independently based on the surface marker expression of the single cells. GO analysis again revealed positive and negative regulators of cell cycle in the non-SuMO and SuMO gene lists, respectively, as well as terms including hematopoietic or lymphoid organ development, immune system development, and hemopoiesis in the non-SuMO list and terms such as response to cytokine stimulus and response to chemical stimulus in the SuMO list (Table S4). Together, these data provide a comprehensive functionally linked gene expression program for single HSCs and provide strong evidence that these genes are central to the HSC self-renewal process. It further provides a paradigm applicable to other stem cell populations for establishing robust cell purification strategies and functional gene expression profiles.

To further confirm the utility of our approach, we next tested index sorting coupled with functional assays in the human mammary system using tissue from patients that had undergone breast reduction surgery. These samples were biologically

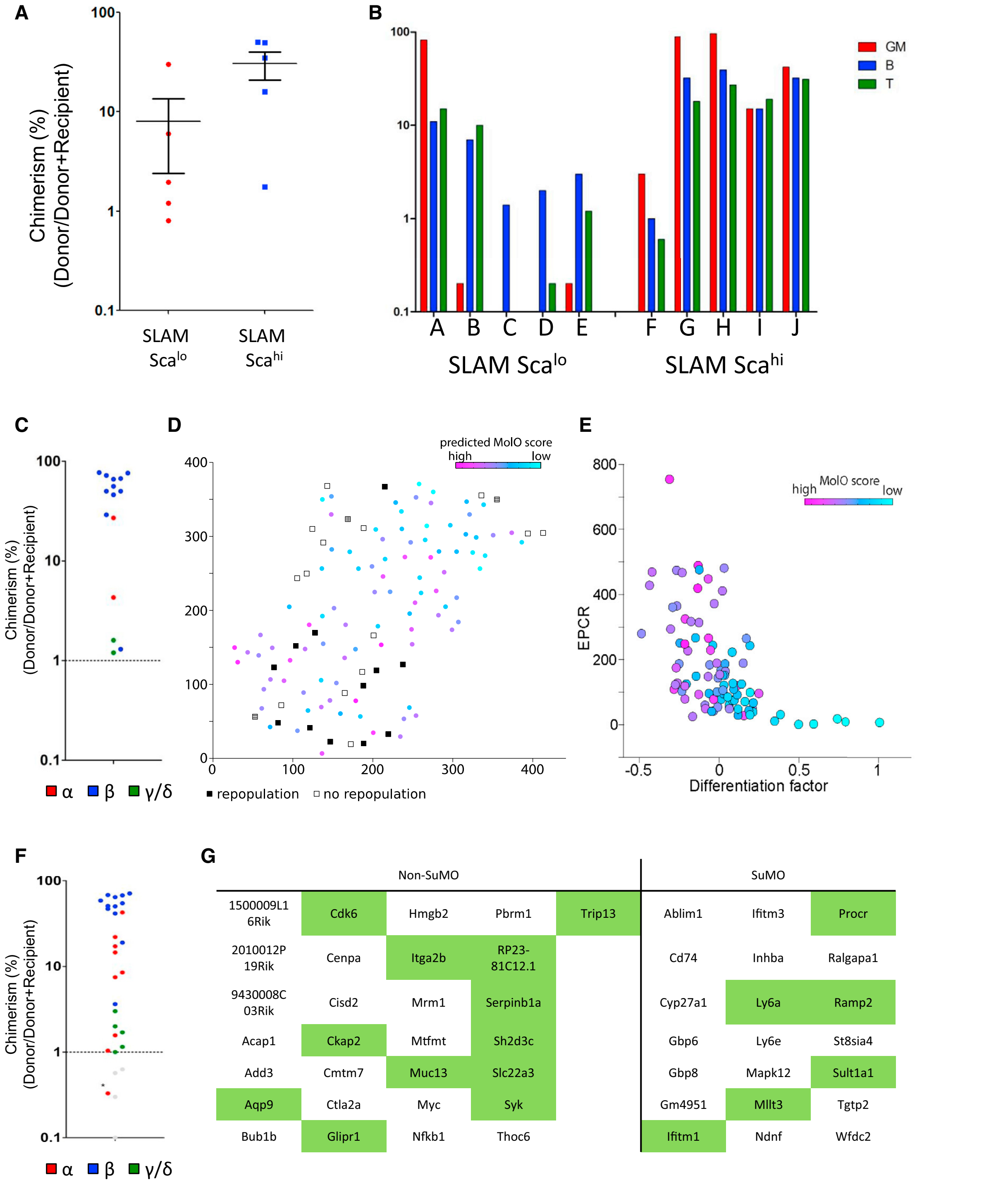

Figure 5. SLAM Sca^{hi} Cells Are Enriched for Long-Term Multilineage HSCs, and Their Single-Cell Transplantation Activity Links to a Distinct Molecular Profile

(A) Donor chimerism (% donor/[% donor + % recipient]) in mice receiving either 10 SLAM Sca^{hi} or SLAM Sca^{lo} cells. Recipients of SLAM Sca^{hi} cells have significantly increased levels of donor chimerism. Error bars represent data ± SEM.

(B) Individual recipient mice of ten SLAM Sca^{hi} or SLAM Sca^{lo} cells and the donor contribution to various lineages. Ratios are formed by taking the total cells of a particular lineage (e.g., GM) and calculating the donor contribution (e.g., Donor GM/(Donor + Recipient GM). GM contribution is red, B is blue, and T is green. Note that four of five recipients of SLAM Sca^{lo} cells have <1% GM contribution, whereas all five recipients of SLAM Sca^{hi} cells have robust myeloid contribution.

(C) Donor chimerism (% donor/[% donor + % recipient]) in mice receiving 1 SLAM Sca^{hi} cell. Fifteen of 29 mice transplanted had donor chimerism of >1% and are displayed on this graph. Blue indicates beta subtype; red indicates alpha subtype; and green indicates gamma/delta subtypes.

(legend continued on next page)

heterogeneous (outbred population, different aged individuals), heavily premanipulated (overnight enzymatic digestion, frozen, and re-thawed), and fewer cell surface markers are used in the purification of progenitor populations. We purified mammary cell progenitors as described (Shehata et al., 2012) and sorted 192 single-cells per patient into individual wells of 96-well culture plates, which were assessed 10–12 days later for the formation of luminal progenitor colonies. Again, we used index sorting to acquire information on forward/side scatter as well as six additional surface markers when sorting the single cells into culture dishes. To permit comparison across patients, we performed z-score normalization of the index sorting results for all patients individually and performed t-SNE on the normalized data. Similar to the hematopoietic data, distinct clusters were resolved (to establish boundaries we performed hierarchical clustering with ward distance, Figure S5C), which were enriched for colony-forming cells for four of five patients (Figure S5D). The average fold increase in colony forming efficiency was 1.6-fold (Figure S5E). To characterize the cluster enriched for colony-forming cells, we performed a Wilcoxon rank sum test to establish that markers were differentially expressed between the identified cluster and the remaining cells. This revealed a significant difference in the fluorescence of five markers, with the largest difference obtained for side scatter (SSC) and EpCAM (Figure S5F). In summary, we used a similar bioinformatic algorithm as in the mouse HSCs to predict that a low SSC and EpCAM^{hi} cell would give rise to a luminal colony and observed enrichment in luminal colony-forming cells in four of the five patients tested. This illustrates the power of combining index sorting and functional outcome in more variable cell systems and sets the stage for other groups to use the technique in their studies to improve purity and link molecular states with functional outcomes.

DISCUSSION

Identifying the molecular regulators of stem cell function has been a long-standing challenge in HSC biology and is complicated due to impurities in isolated populations and, more recently, the identification of functional heterogeneity in HSCs themselves. Because the assignment of HSC status relies on retrospective assays measuring their progeny, the transcriptome of the original HSC is no longer accessible. Therefore, if one is to identify the individual molecules and the regulatory networks at play within these cellular systems, alternative approaches are required. Using a combination of single-cell functional assays and single-cell gene expression linked together by flow cytometric index sorting, we provide insights into the gene expression program of transplantable multilineage HSCs compared with fluorescence-activated cell sorting (FACS)-marker-defined HSCs that lack HSC activity.

A number of laboratories have refined strategies to isolate enriched populations of HSCs with functional purities of up to ~50% (Beerman et al., 2010; Dykstra et al., 2007; Goodell et al., 1996; Morita et al., 2010). While each strategy identifies functional HSCs, they do not share the same cell surface markers in many cases. We took advantage of this diversity, assuming that each strategy contained both HSCs and non-HSCs and that the HSCs would share a common molecular program. Utilizing four distinct isolation strategies, we were able to identify common gene expression patterns within HSC populations (MolO HSCs), which featured numerous genes previously implicated in HSC biology (e.g., Gata2, Gfi1b, and vWF, Figure 1) and also reveal previously unrecognized potential players in HSC biology.

MolO HSCs were further distinguishable by higher than average CD150 and Sca-1 expression as well as lower than average CD48 expression. These cell surface markers had been previously implicated to be of significant importance with CD150^{high} cells enriching for HSCs with greater self-renewal (Beerman et al., 2010; Morita et al., 2010), and while genetic ablation of Sca-1 had no impact on HSC self-renewal (Bradfute et al., 2005), lower SP^{KLS} cells were shown to have a higher Sca-1 expression (Challen et al., 2010). However, no previous studies have used very bright Sca-1 in combination with CD150 to define an HSC population, which our molecular overlapping study predicted to be very effective at isolating near pure HSCs. Using the cell surface expression of MolO HSCs compared with NoMO HSCs, we could take an unbiased approach to identify the simplest and most discriminating combination of markers. The resultant SLAM Sca^{hi} population was indeed greatly enriched for HSCs compared with the SLAM Sca^{lo}, despite the SLAM Sca^{lo} cells still expressing Sca-1 as well as the vast majority of other popular HSC markers. We further refined our HSC isolation strategy based on a recently published bioinformatic analysis, scLVM (Buettner et al., 2015), and report a single-cell long term multilineage efficiency of 67% using the EPCR^{hi}SLAMSca^{hi} phenotype. It is likely that technical challenges of the single-cell transplantation assay will limit its efficiency, and future studies are needed to evaluate whether efficiencies higher than ~70% can be obtained. Of note, our dataset may also be used to determine genes unlikely to be expressed in functional HSCs, which may help identifying specific contaminating cells from within each specific FACS-maker-defined HSC population.

Our data report a genome-wide gene expression dataset for single HSCs suitable to separate out the most likely functional

(D) Joint representation of sequenced cells and transplanted cells. In the t-SNE space, cells with a high predicted MolO score cluster together with repopulating cells; cells with a low predicted MolO score cluster with mostly non-repopulators. Transplanted cells are represented by squares. White indicates non-repopulators. Black indicates repopulators. Hatch pattern indicates gamma-HSCs and the 1% chimerism beta-HSC highlighted in the main text. Sequenced cells are represented by circles, and the predicted MolO score is shown. Axes are in arbitrary units.

(E) The hidden differentiation factor recovered using scLVM was strongly correlated with EPCR expression. Cells with high EPCR expression and low differentiation factor also had a high predicted MolO score (colors as in D). Axes are in arbitrary units.

(F) Donor chimerism (% donor/[% donor + % recipient]) in mice receiving 1 ESLAM Sca^{hi} cell. Twenty-six of 39 mice transplanted had donor chimerism of >1% and are displayed on this graph. Blue indicates beta subtype. Red indicates alpha subtype, and green indicates gamma/delta subtypes. The asterisk indicates an HSC that had <1% chimerism at 16 weeks, but >1% at 24 weeks.

(G) Table of signature genes significantly associated with SuMO and non-SuMO cells. Overlapping genes with the MolO/NoMO gene list are highlighted in green. See also Figure S5 and Table S4.

HSCs. The most pronounced difference in terms of gene expression is the significant enrichment of genes, which are involved in cell cycle, where the NoMO cells are primed toward proliferation, whereas the MolO cells express high levels of cell-cycle inhibitors. This genetic signature is supported by in vitro data demonstrating that single SLAM Sca^{lo} cells (enriched for NoMO) were significantly more proliferative compared with the SLAM Sca^{hi} cells (enriched for MolO). These data support the idea that SLAM Sca^{hi}/MolO cells might constitute the long-term reservoir of dormant HSCs that respond to stress or injury (Ohlstein et al., 2004; Wilson et al., 2008).

The integration of the genome-wide scRNA-seq analysis with the index sorting data also suggests that while functional HSCs are typically dormant, they have the ability to respond to extrinsic signaling for stress and injury (King and Goodell, 2011; Wilson et al., 2008), further supporting their robust activity in long-term transplantation assays. Linking molecular signatures to functional activity is one of the most challenging aspects of stem cell biology. Overlaying our single-cell transplantation data onto the scRNA-seq data allowed us to separate the non-repopulating HSC from the repopulating HSCs, offering insights into the molecular programs that define a repopulating HSC. Of note, our data focus on HSCs that read out in a transplantation assay with direct relevance to the therapeutic potential of HSCs, but investigation of the control mechanisms underlying steady-state hematopoiesis is likely to require different experimental strategies (Busch et al., 2015). Our approach can be extended in future to study the molecular programs of individual lineage-biased HSCs and HSCs with durable compared with finite self-renewal. Linking gene expression changes with functional data through index sorting establishes an experimental paradigm that can be exploited in any cell population with a reasonably high purity and defined single-cell functional assays. This will greatly enhance studies of normal and malignant blood stem cells, as well as those in other cellular systems such as mammary and neural stem cells.

EXPERIMENTAL PROCEDURES

Detailed experimental protocols are provided in Supplemental Experimental Procedures.

Purification of Stem and Progenitor Cells

Suspensions of bone marrow (BM) cells from the femurs, tibiae, and iliac crest of 8- to 12-week-old C57BL/6 mice were isolated and depleted of red blood cells by an ammonium chloride lysis step (STEMCELL Technologies). Antibodies for HSC isolation are listed in Supplemental Experimental Procedures. Cells were sorted using a Becton Dickinson Influx sorter equipped with five lasers. For single-cell gene expression assays, cells were sorted into individual wells of 96-well PCR plates. For single-cell transplantation and in vitro assays, cells were sorted into individual wells of a U-bottom 96-well plate. For progenitor colony forming cell assays and ten-cell transplantation assays, cells were sorted into 1.5-ml tubes containing serum-free medium.

Progenitor Cell Assays

Five hundred CMPs, MEPs, or GMPs were sorted into serum-free medium, divided into a high concentration fraction (~450 cells) and a low concentration fraction (~45 cells), placed into semisolid medium containing myeloid growth factors (MC3434; STEMCELL), and counted after 10 and 14 days of culture. Single LMPPs were sorted into wells containing OP9 cells supplemented with 100 ng/ml interleukin-7 (IL-7) and 50 ng/ml FLT-3, harvested at day 28 and analyzed for the presence of B (defined as B220$^+$) and myeloid (Ly6g$^+$ and/or Mac1$^+$) cells.

Single HSC Cultures

SLAM Sca^{hi} and SLAM Sca^{lo} HSCs were sorted and cultured in STEMSPAN medium containing SCF and IL-11 as described previously (Kent et al., 2008, 2013). After 24 hr, wells were scored for the presence of a single cell and counted each day to track the clonal growth of individual cells. For immunophenotyping, clones were individually stained and assessed for the expression of Sca-1, c-Kit, and a panel of lineage markers along with 7-amino-actinomycin D (7AAD, Invitrogen) to mark dead cells.

Clone Size Calculations and Antibody Information for In Vitro Cultures

Clones were estimated to be small (50–5,000 cells), medium (5,000–20,000 cells), or large (20,000 or more cells). No clones had fewer than 50 cells. Ten-day clones were stained with biotinylated lineage marker antibodies (Haematopoietic Progenitor Enrichment Cocktail; STEMCELL), c-Kit, and Sca-1. Cells were enumerated using a defined number of fluorescent beads (Trucount Control Beads, Becton Dickinson).

Single-Cell Gene Expression Analysis

Single-cell gene expression analysis was performed as described previously (Moignard et al., 2013). Single-cell expression data were collected using the Fluidigm Data Collection software. ΔCt values were calculated as previously described (Guo et al., 2010) by cell-wise normalization to the mean expression level of two housekeeping genes (Ubc and Polr2a). All housekeepers, Cdkn2a and Egfl7 were removed from the dataset for downstream analysis. Cdkn2a was not expressed in any of the cell types, and Egfl7 assay experienced technical issues. Hierarchical clustering was performed in R (http://www.r-project.org) using the hclust package and heatmap.2 from the gplots package using Spearman rank correlations and ward linkage. t-SNE was performed in Matlab (Mathworks) using the Matlab implementation (http://homepage.tudelft.nl/19j49/t-SNE.html) with standard settings.

We identified MolO cells based on a weighting matrix defined by repopulation probabilities and the 2D t-SNE representation of the data (Figures S2B and S2C). Random forests were trained on the normalized Ct values of the set of genes, which were assayed by single-cell gene expression and variable above technical noise in scRNA-seq. Training was performed on all cells from sorting strategy HSC1, and generalizability was quantified using 10-fold cross-validation (Figure S3C). Training and testing of the classifier was performed in python 2.7 using the sklearn library.

scRNA-Seq

scRNA-seq analysis was performed as described previously (Picelli et al., 2014). Single cells were sorted by FACS directly into individual wells of a 96-well plate containing lysis buffer, and libraries were prepared using the Illumina Nextera XT DNA preparation kit. Pooled libraries were run on the Illumina Hi-Seq 2500 and reads aligned using STAR (Dobin et al., 2013). HTSeq (Anders et al., 2014) was run to assign mapped reads to Ensembl genes. Mapped reads were normalized using size factors as described (Brennecke et al., 2013). We estimated technical noise (Brennecke et al., 2013) and fitted the relation between mean read counts and squared coefficient of variation using ERCC spike-ins (Life Technologies) (Figure 3Ai). Genes for which the squared coefficient of variation exceeded technical noise were considered variable.

Transplantation of HSCs

Ten-cell transplantations were performed in CD45.1 lethally irradiated C57Bl/6 recipients along with 250,000 spleen CD45.1/.2 helper cells. Single-cell transplantations were performed by tail vein injection of sublethally irradiated Ly5-congenic adult W41/W41 mice as previously described (Dykstra et al., 2007). Peripheral blood samples were collected from the tail vein of several mice at 4 weeks and all mice at 8, 16, and 24 weeks after transplantation. Donor and recipient cells were distinguished by their expression of CD45.1 or CD45.2. Animals with at least 1% donor white blood cells (WBCs) at 16 and/or 24 weeks after transplantation were considered to be repopulated with long-term reconstituting cells. HSCs were further discriminated according to previously described high (alpha or beta) or low (gamma or delta) ratios of their proportional contributions to the GM, B cell, and T cell subsets at 16 weeks after transplantation (Dykstra et al., 2007).

Isolation and Assessment of Mammary Progenitors

All primary human material was derived from five reduction mammoplasties at Addenbrooke's Hospital under full informed consent and in accordance with the National Research Ethics Service, Cambridgeshire 2 Research Ethics Committee approval (08/H0308/178) as part of the Adult Breast Stem Cell Study. All tissue donors had no previous history of cancer and were premenopausal (ages 20 to 23). Mammary tissue was dissociated to single-cell suspensions as previously described (Eirew et al., 2010). Single-cell suspensions of human mammary cells were treated to detect the enzyme activity of aldehyde dehydrogenase (ALDH) using the Aldefluor Kit (StemCell Technologies) as per the manufacturer's instructions. Antibodies for mammary progenitor cell isolation are listed in Supplemental Experimental Procedures. Cells were sorted using a Becton Dickinson Influx. Luminal progenitor populations were seeded as single cells into 96-well plates with 1×10^4 irradiated NIH 3T3 feeder cells. Cultures were maintained in Human EpiCult-B (StemCell Technologies) supplemented with 5% fetal bovine serum (FBS) (StemCell Technologies) and 50 μg/ml gentamicin for 10 to 12 days.

Mice

C57Bl/6J (B6)-Ly5.2 mice and congenic B6-W41/W41-Ly5.1 (W41-5.1) mice were bred and maintained at the University of Cambridge in microisolator cages and provided continuously with sterile food, water, and bedding. All mice were kept in specified pathogen-free conditions, and all procedures were performed according to the United Kingdom Home Office regulations.

ACCESSION NUMBERS

RNA-seq data for HSC1 have been deposited into the NCBI GEO portal under the accession number GEO: GSE61533.

SUPPLEMENTAL INFORMATION

Supplemental Information includes Supplemental Experimental Procedures, five figures, and four tables and can be found with this article online at http://dx.doi.org/10.1016/j.stem.2015.04.004.

AUTHOR CONTRIBUTIONS

Experiments were designed by D.G.K., N.K.W., and B.G. HSC and progenitor cell isolation was performed by D.G.K., N.K.W., and R.S., with assistance from F.J.C.-N. Clonal progenitor assays were performed by D.G.K. with assistance from N.K.W. D.G.K. and C.A.O. performed homing and cell-cycle assays. M.S. performed mammary isolation and colony assays. Single-cell Fluidigm gene expression profiling was performed by N.K.W. with assistance from F.J.C.-N. Single-cell and bulk transplantation assays were carried out by D.G.K. Single-cell HSC assays and flow cytometry analysis were performed by D.G.K. with assistance from N.K.W. Clustering and multidimensional analyses of Fluidigm data was done primarily by F.B. with assistance from N.K.W. and M.S.C. scRNA-seq pipeline was developed by I.C.M., T.V., and C.P.P. scRNA-seq libraries were prepared by I.C.M. RNA-seq analysis was performed by F.B., E.D., and N.K.W. Multidimensional analysis of cell surface marker and RNA-seq data was performed by F.B. with input from N.K.W., D.G.K., B.G., and F.J.T. T.V. and C.P.P. contributed to the single-cell RNA-seq pipeline. J.S. and C.C. provided samples and mammary cell experimental assistance, and A.R.G. supported the establishment of transplantation assays. N.K.W., D.G.K., F.B., and B.G. wrote the paper with input from F.J.T., M.S., J.S., and A.R.G. B.G. directed the research.

ACKNOWLEDGMENTS

We thank Chiara Cossetti and Michal Maj at the CIMR Flow Cytometry Core for their help with cell sorting, Dean Pask and Tina Hamilton for technical assistance, Vicki Moignard for advice on Fluidigm Biomark analyses, and Cheuk-Ho Tsang for helpful suggestions. Work in the author's laboratory is supported by grants from the Leukaemia and Lymphoma Research, the Medical Research Council, Cancer Research UK, Biotechnology and Biological Sciences Research Council, Leukemia Lymphoma Society, and the National Institute for Health Research Cambridge Biomedical Research Centre and core support grants by the Wellcome Trust to the Cambridge Institute for Medical Research and Wellcome Trust-MRC Cambridge Stem Cell Institute. D.G.K. is the recipient of a Canadian Institutes of Health Research Postdoctoral Fellowship. F.B. and F.J.T. are funded by the European Research Council (starting grant "LatentCauses"). For funding for the open access charge, the core support grant was provided by the Wellcome Trust-MRC Cambridge Stem Cell Institute. We acknowledge the support of the University of Cambridge, Cancer Research UK Institute (core grant C14303/A17197), and Hutchison Whampoa Limited.

Received: October 9, 2014
Revised: February 26, 2015
Accepted: April 10, 2015
Published: May 21, 2015

REFERENCES

Adolfsson, J., Borge, O.J., Bryder, D., Theilgaard-Mönch, K., Astrand-Grundström, I., Sitnicka, E., Sasaki, Y., and Jacobsen, S.E. (2001). Upregulation of Flt3 expression within the bone marrow Lin(-)Sca1(+)c-kit(+) stem cell compartment is accompanied by loss of self-renewal capacity. Immunity *15*, 659–669.

Adolfsson, J., Månsson, R., Buza-Vidas, N., Hultquist, A., Liuba, K., Jensen, C.T., Bryder, D., Yang, L., Borge, O.J., Thoren, L.A., et al. (2005). Identification of Flt3+ lympho-myeloid stem cells lacking erythro-megakaryocytic potential a revised road map for adult blood lineage commitment. Cell *121*, 295–306.

Akashi, K., Traver, D., Miyamoto, T., and Weissman, I.L. (2000). A clonogenic common myeloid progenitor that gives rise to all myeloid lineages. Nature *404*, 193–197.

Amir, el-A.D., Davis, K.L., Tadmor, M.D., Simonds, E.F., Levine, J.H., Bendall, S.C., Shenfeld, D.K., Krishnaswamy, S., Nolan, G.P., and Pe'er, D. (2013). viSNE enables visualization of high dimensional single-cell data and reveals phenotypic heterogeneity of leukemia. Nat Biotechnol *31*, 545–552.

Anders, S., Pyl, P.T., and Huber, W. (2014). HTSeq—A Python framework to work with high-throughput sequencing data. Bioinformatics *31*, 166–169.

Beerman, I., Bhattacharya, D., Zandi, S., Sigvardsson, M., Weissman, I.L., Bryder, D., and Rossi, D.J. (2010). Functionally distinct hematopoietic stem cells modulate hematopoietic lineage potential during aging by a mechanism of clonal expansion. Proc. Natl. Acad. Sci. USA *107*, 5465–5470.

Bradfute, S.B., Graubert, T.A., and Goodell, M.A. (2005). Roles of Sca-1 in hematopoietic stem/progenitor cell function. Exp. Hematol. *33*, 836–843.

Breiman, L. (2001). Random Forests. Mach. Learn. *45*, 5–32.

Brennecke, P., Anders, S., Kim, J.K., Kołodziejczyk, A.A., Zhang, X., Proserpio, V., Baying, B., Benes, V., Teichmann, S.A., Marioni, J.C., and Heisler, M.G. (2013). Accounting for technical noise in single-cell RNA-seq experiments. Nat. Methods *10*, 1093–1095.

Bryder, D., Rossi, D.J., and Weissman, I.L. (2006). Hematopoietic stem cells: the paradigmatic tissue-specific stem cell. Am. J. Pathol. *169*, 338–346.

Buettner, F., Natarajan, K.N., Casale, F.P., Proserpio, V., Scialdone, A., Theis, F.J., Teichmann, S.A., Marioni, J.C., and Stegle, O. (2015). Computational analysis of cell-to-cell heterogeneity in single-cell RNA-sequencing data reveals hidden subpopulations of cells. Nat. Biotechnol. *33*, 155–160.

Busch, K., Klapproth, K., Barile, M., Flossdorf, M., Holland-Letz, T., Schlenner, S.M., Reth, M., Höfer, T., and Rodewald, H.R. (2015). Fundamental properties of unperturbed haematopoiesis from stem cells in vivo. Nature *518*, 542–546.

Challen, G.A., Boles, N.C., Chambers, S.M., and Goodell, M.A. (2010). Distinct hematopoietic stem cell subtypes are differentially regulated by TGF-beta1. Cell Stem Cell *6*, 265–278.

Copley, M.R., and Eaves, C.J. (2013). Developmental changes in hematopoietic stem cell properties. Exp. Mol. Med. *45*, e55.

Dobin, A., Davis, C.A., Schlesinger, F., Drenkow, J., Zaleski, C., Jha, S., Batut, P., Chaisson, M., and Gingeras, T.R. (2013). STAR: ultrafast universal RNA-seq aligner. Bioinformatics *29*, 15–21.

Dykstra, B., Kent, D., Bowie, M., McCaffrey, L., Hamilton, M., Lyons, K., Lee, S.J., Brinkman, R., and Eaves, C. (2007). Long-term propagation of distinct hematopoietic differentiation programs in vivo. Cell Stem Cell *1*, 218–229.

Eirew, P., Stingl, J., and Eaves, C.J. (2010). Quantitation of human mammary epithelial stem cells with in vivo regenerative properties using a subrenal capsule xenotransplantation assay. Nat. Protoc. *5*, 1945–1956.

Ema, H., Morita, Y., and Suda, T. (2014). Heterogeneity and hierarchy of hematopoietic stem cells. Exp. Hematol. *42*, 74–82.e72.

Goodell, M.A., Brose, K., Paradis, G., Conner, A.S., and Mulligan, R.C. (1996). Isolation and functional properties of murine hematopoietic stem cells that are replicating in vivo. J. Exp. Med. *183*, 1797–1806.

Grinenko, T., Arndt, K., Portz, M., Mende, N., Günther, M., Cosgun, K.N., Alexopoulou, D., Lakshmanaperumal, N., Henry, I., Dahl, A., and Waskow, C. (2014). Clonal expansion capacity defines two consecutive developmental stages of long-term hematopoietic stem cells. J. Exp. Med. *211*, 209–215.

Guo, G., Huss, M., Tong, G.Q., Wang, C., Li Sun, L., Clarke, N.D., and Robson, P. (2010). Resolution of cell fate decisions revealed by single-cell gene expression analysis from zygote to blastocyst. Dev. Cell *18*, 675–685.

Kent, D.G., Dykstra, B.J., Cheyne, J., Ma, E., and Eaves, C.J. (2008). Steel factor coordinately regulates the molecular signature and biologic function of hematopoietic stem cells. Blood *112*, 560–567.

Kent, D.G., Copley, M.R., Benz, C., Wöhrer, S., Dykstra, B.J., Ma, E., Cheyne, J., Zhao, Y., Bowie, M.B., Zhao, Y., et al. (2009). Prospective isolation and molecular characterization of hematopoietic stem cells with durable self-renewal potential. Blood *113*, 6342–6350.

Kent, D.G., Li, J., Tanna, H., Fink, J., Kirschner, K., Pask, D.C., Silber, Y., Hamilton, T.L., Sneade, R., Simons, B.D., and Green, A.R. (2013). Self-renewal of single mouse hematopoietic stem cells is reduced by JAK2V617F without compromising progenitor cell expansion. PLoS Biol. *11*, e1001576.

Kiel, M.J., Yilmaz, O.H., Iwashita, T., Yilmaz, O.H., Terhorst, C., and Morrison, S.J. (2005). SLAM family receptors distinguish hematopoietic stem and progenitor cells and reveal endothelial niches for stem cells. Cell *121*, 1109–1121.

Kiel, M.J., Radice, G.L., and Morrison, S.J. (2007). Lack of evidence that hematopoietic stem cells depend on N-cadherin-mediated adhesion to osteoblasts for their maintenance. Cell Stem Cell *1*, 204–217.

King, K.Y., and Goodell, M.A. (2011). Inflammatory modulation of HSCs: viewing the HSC as a foundation for the immune response. Nat. Rev. Immunol. *11*, 685–692.

Månsson, R., Hultquist, A., Luc, S., Yang, L., Anderson, K., Kharazi, S., Al-Hashmi, S., Liuba, K., Thorén, L., Adolfsson, J., et al. (2007). Molecular evidence for hierarchical transcriptional lineage priming in fetal and adult stem cells and multipotent progenitors. Immunity *26*, 407–419.

Moignard, V., Macaulay, I.C., Swiers, G., Buettner, F., Schütte, J., Calero-Nieto, F.J., Kinston, S., Joshi, A., Hannah, R., Theis, F.J., et al. (2013). Characterization of transcriptional networks in blood stem and progenitor cells using high-throughput single-cell gene expression analysis. Nat. Cell Biol. *15*, 363–372.

Morita, Y., Ema, H., and Nakauchi, H. (2010). Heterogeneity and hierarchy within the most primitive hematopoietic stem cell compartment. J. Exp. Med. *207*, 1173–1182.

Naik, S.H., Perié, L., Swart, E., Gerlach, C., van Rooij, N., de Boer, R.J., and Schumacher, T.N. (2013). Diverse and heritable lineage imprinting of early haematopoietic progenitors. Nature *496*, 229–232.

Ohlstein, B., Kai, T., Decotto, E., and Spradling, A. (2004). The stem cell niche: theme and variations. Curr. Opin. Cell Biol. *16*, 693–699.

Osborne, G.W. (2011). Recent advances in flow cytometric cell sorting. Methods Cell Biol. *102*, 533–556.

Picelli, S., Faridani, O.R., Björklund, A.K., Winberg, G., Sagasser, S., and Sandberg, R. (2014). Full-length RNA-seq from single cells using Smart-seq2. Nat. Protoc. *9*, 171–181.

Prick, J., de Haan, G., Green, A.R., and Kent, D.G. (2014). Clonal heterogeneity as a driver of disease variability in the evolution of myeloproliferative neoplasms. Exp. Hematol. *42*, 841–851.

Rieger, M.A., Hoppe, P.S., Smejkal, B.M., Eitelhuber, A.C., and Schroeder, T. (2009). Hematopoietic cytokines can instruct lineage choice. Science *325*, 217–218.

Sanjuan-Pla, A., Macaulay, I.C., Jensen, C.T., Woll, P.S., Luis, T.C., Mead, A., Moore, S., Carella, C., Matsuoka, S., Bouriez Jones, T., et al. (2013). Platelet-biased stem cells reside at the apex of the haematopoietic stem-cell hierarchy. Nature *502*, 232–236.

Shehata, M., Teschendorff, A., Sharp, G., Novcic, N., Russell, I.A., Avril, S., Prater, M., Eirew, P., Caldas, C., Watson, C.J., and Stingl, J. (2012). Phenotypic and functional characterisation of the luminal cell hierarchy of the mammary gland. Breast Cancer Res. *14*, R134.

van der Maaten, L., and Hinton, G. (2008). Visualizing Data using t-SNE. J. Mach. Learn. Res. *9*, 2579–2605.

Weksberg, D.C., Chambers, S.M., Boles, N.C., and Goodell, M.A. (2008). CD150- side population cells represent a functionally distinct population of long-term hematopoietic stem cells. Blood *111*, 2444–2451.

Wilkinson, A.C., and Göttgens, B. (2013). Transcriptional regulation of haematopoietic stem cells. Adv. Exp. Med. Biol. *786*, 187–212.

Wilson, A., Laurenti, E., Oser, G., van der Wath, R.C., Blanco-Bose, W., Jaworski, M., Offner, S., Dunant, C.F., Eshkind, L., Bockamp, E., et al. (2008). Hematopoietic stem cells reversibly switch from dormancy to self-renewal during homeostasis and repair. Cell *135*, 1118–1129.

Yamamoto, R., Morita, Y., Ooehara, J., Hamanaka, S., Onodera, M., Rudolph, K.L., Ema, H., and Nakauchi, H. (2013). Clonal analysis unveils self-renewing lineage-restricted progenitors generated directly from hematopoietic stem cells. Cell *154*, 1112–1126.

Cell Stem Cell
Supplemental Information

Combined Single-Cell Functional and Gene Expression Analysis Resolves Heterogeneity within Stem Cell Populations

Nicola K. Wilson, David G. Kent, Florian Buettner, Mona Shehata, Iain C. Macaulay, Fernando J. Calero-Nieto, Manuel Sánchez Castillo, Caroline A. Oedekoven, Evangelia Diamanti, Reiner Schulte, Chris P. Ponting, Thierry Voet, Carlos Caldas, John Stingl, Anthony R. Green, Fabian J. Theis, and Berthold Göttgens

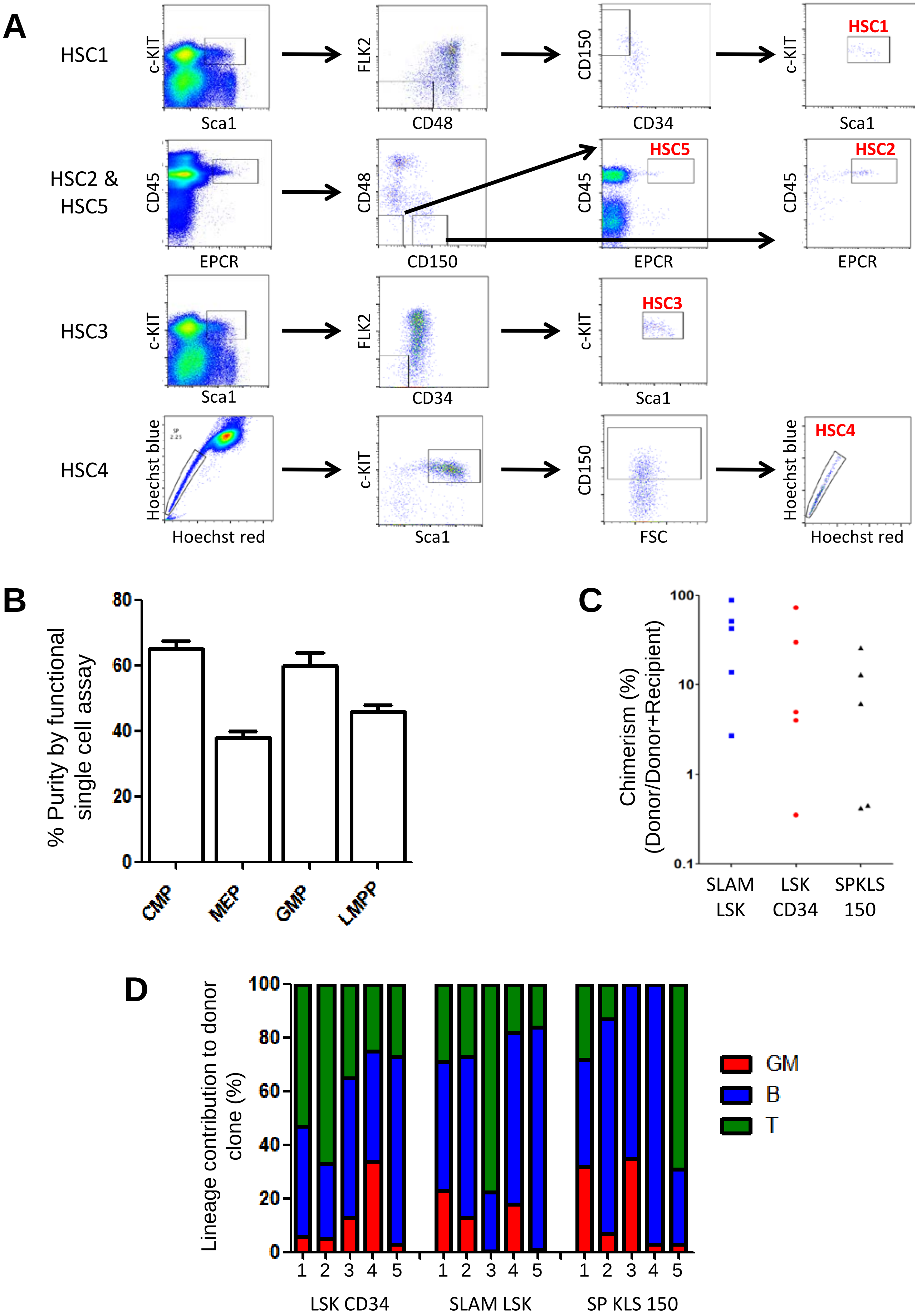

Wilson et al Figure S1

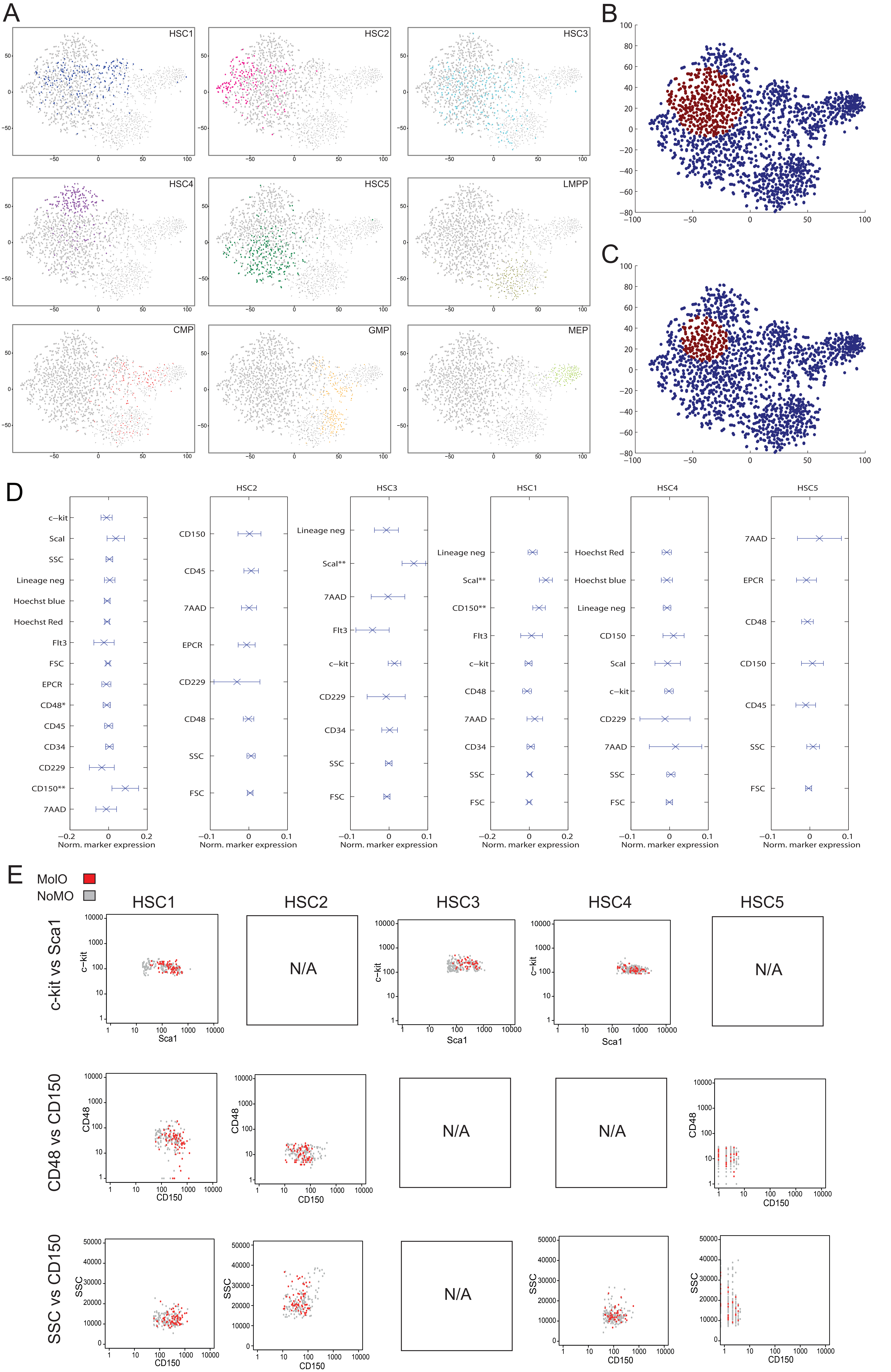

Wilson et al Figure S2

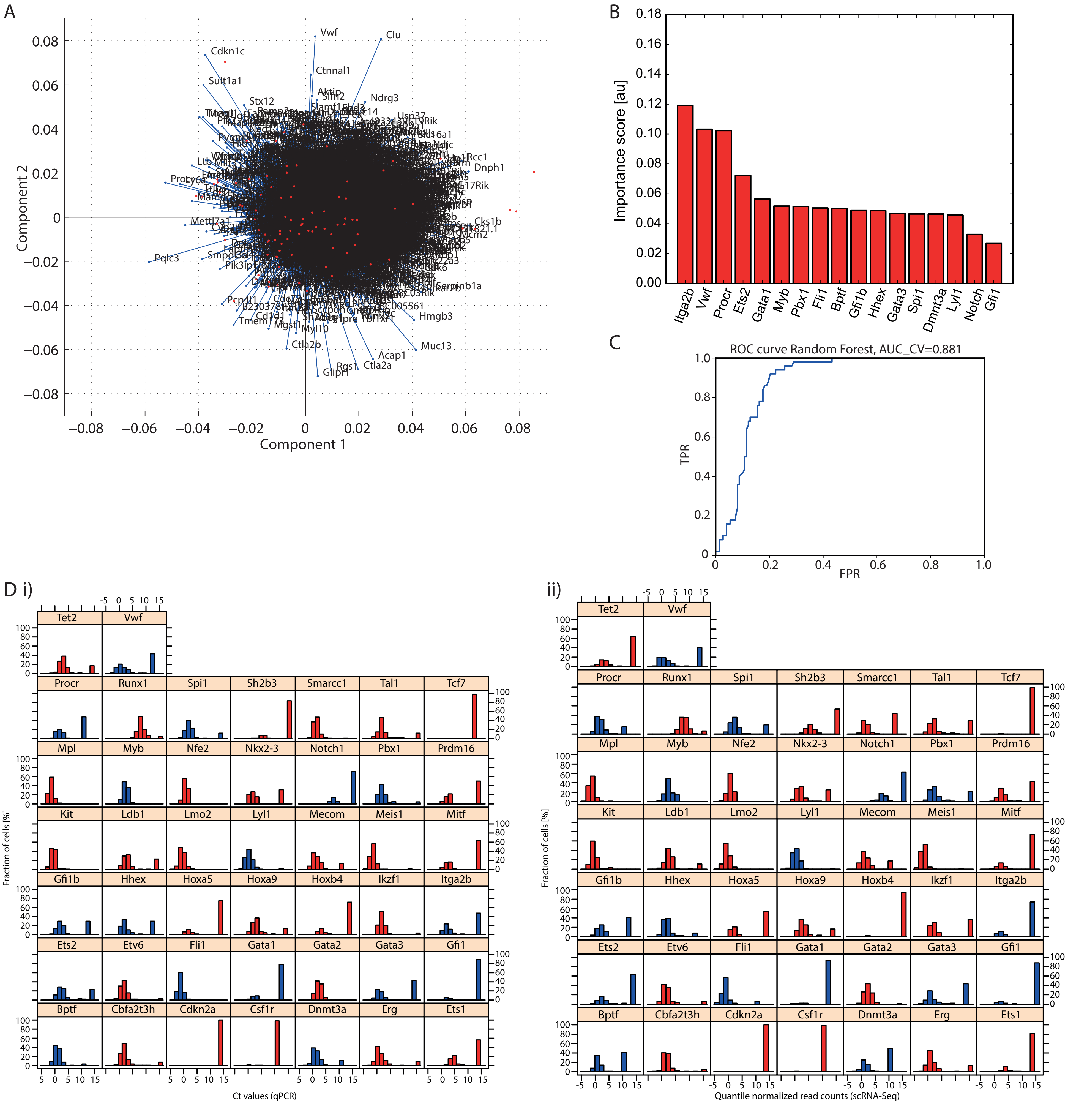

Wilson et al Figure S3

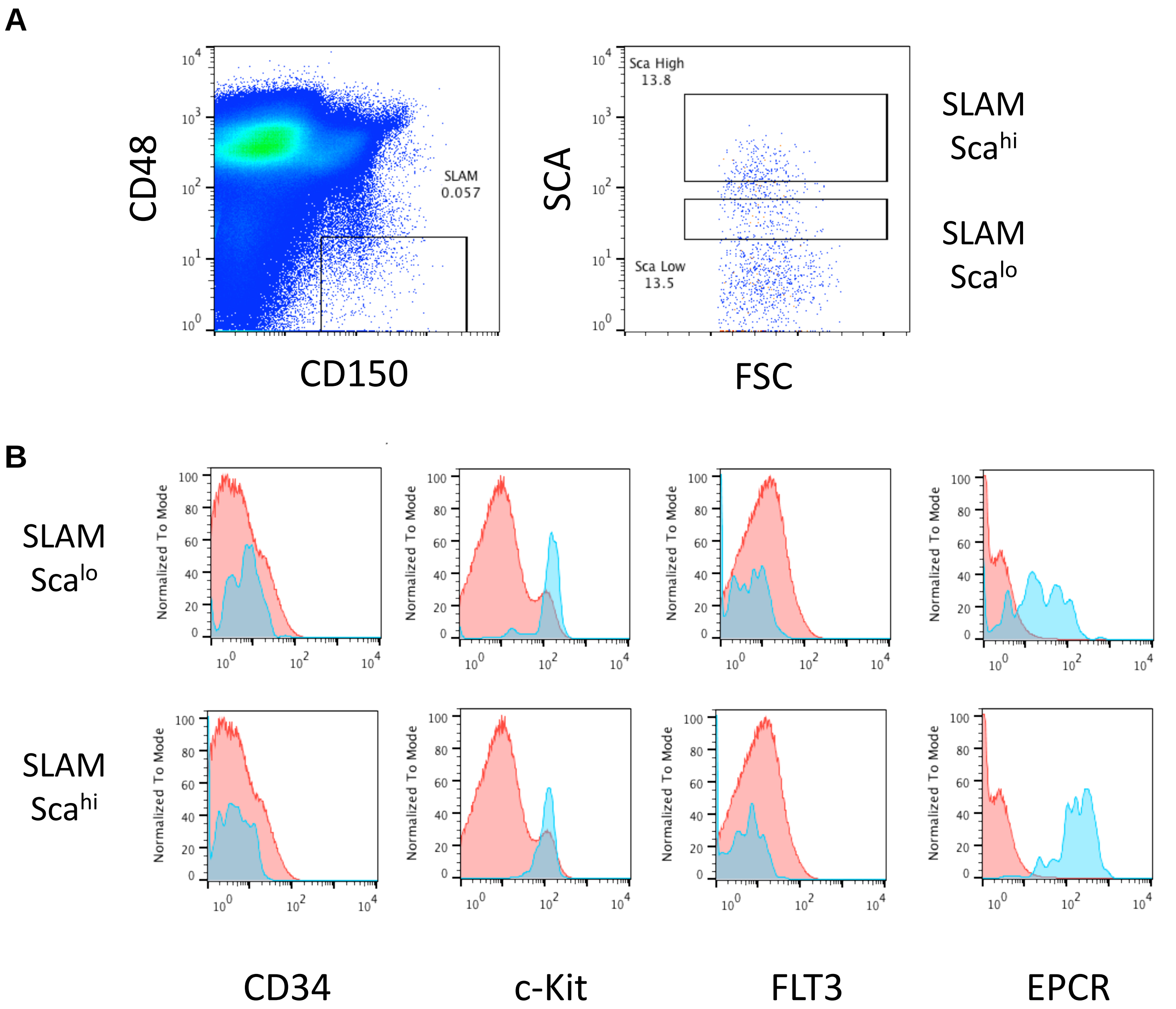

Wilson et al Figure S4

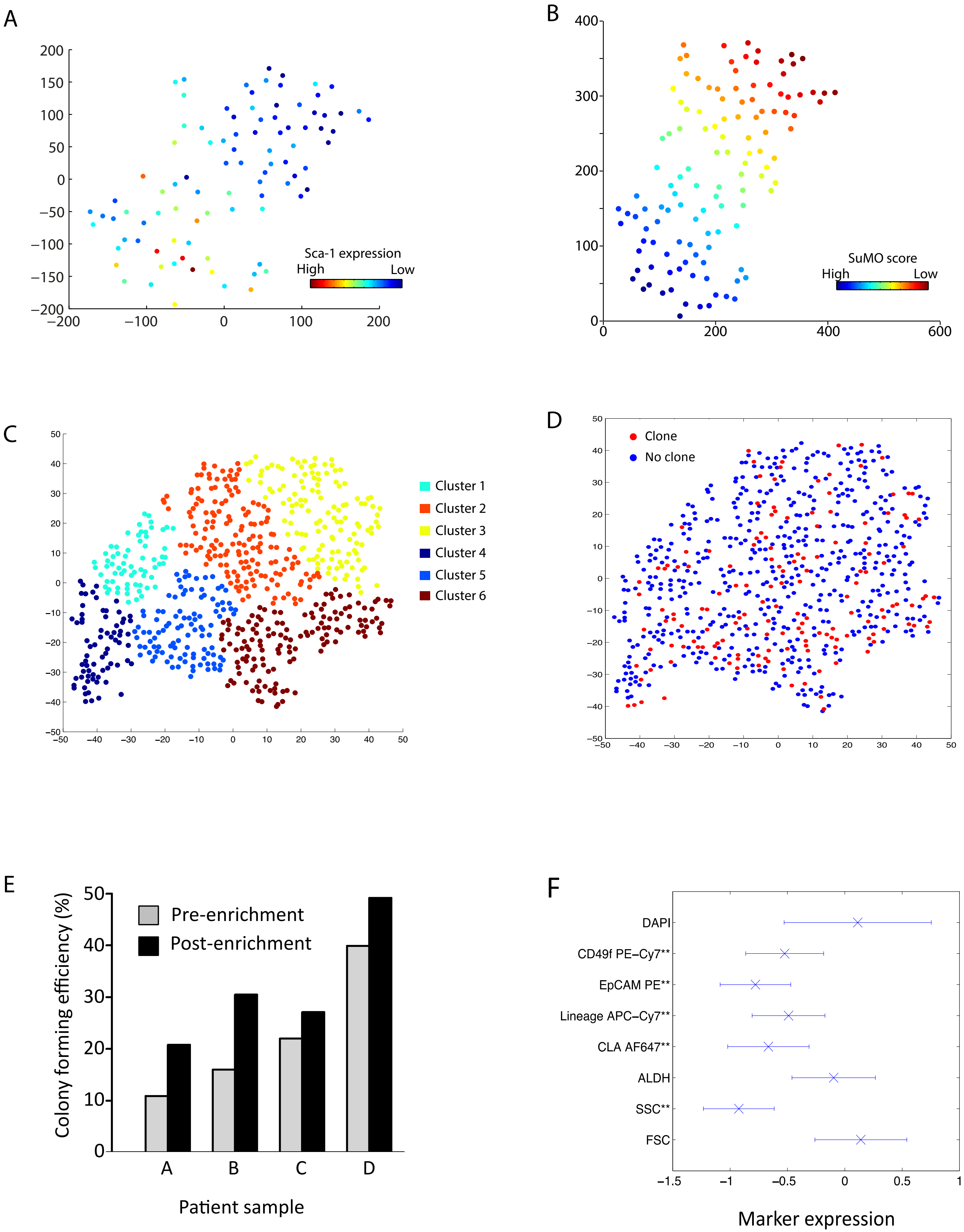

Wilson et al Figure S5

Supplemental Information

Figure S1, related to Figure 1; Functional validation of sorted populations for gene expression profiling.

A) Sorting and gating strategies for the isolation of all HSC populations. B) Individual progenitor cells were isolated and seeded in a methylcellulose-based colony assay. For CMP, GMP and MEPs, 500 cells were isolated and placed into methylcellulose cultures. Colonies were morphologically scored as mixed, erythroid, or granulocytic/monocytic and the percentage of the correct colony type (e.g., granulocyte colony for GMP) is depicted. For LMPPs, single cells were sorted onto OP9 feeder cells and scored for the presence of myeloid and lymphoid elements. The percentage purity is indicated on the y axis. Error bars represent data ± SEM. C) Each individual dot represents the donor chimerism at 16 weeks in a recipient of 10 phenotypically identified HSCs. The phenotypes are indicated on the x-axis and the % donor contribution on the y-axis. D) The stacked bar graphs depict the relative contribution of the donor clone to each of the granulocyte/monocyte (GM, red), B-cell (B, blue), and T-cell (T, green) lineages.

Figure S2, related to Figure 2; Robustness analysis.

t-SNE plot of all cells calculated from the 43 genes analysed by Fluidigm. Axes are in arbitrary units. A) Individual populations are shown for clarity. B-C) t-SNE plot with the MolO HSCs identified by the computational weighting highlighted in red. B) Leeway factor of 70%. C) Leeway factor of 90%. D) Difference in normalised marker expression between MolO cells and NoMO cells for each sorting strategy. Error bars correspond to 1 standard deviation. First column represents all MolO cells compared to all other HSCs across sorting

strategies. * = p<0.05, ** = p<0.01. E) FACs plots for the individual HSC sorting strategies, showing c-kit, Sca-1, CD48 and CD150. MolO HSCs are highlighted in red, NoMO HSCs are highlighted in gray.

Figure S3, related to Figure 3; RNA-Seq analysis

A) Principal component loading plot, indicating the 4533 differentially regulated genes identified from the scRNA-Seq. The 92 single cells which were analysed by scRNA-Seq are shown within the plot as red circles. B) Importance of the individual genes for predicting MolO cells using random forests, quantified as the total decrease in node impurity averaged over all trees in the random forest (gini importance). C) ROC curve quantifying classification performance and generalizability of MolO predictor using 10-fold cross-validation. An area under the cross-validated ROC curve of 0.881 indicates that in 88% percent of predictions, a randomly drawn MolO cell would receive a higher MolO score than a randomly drawn NoMO cell. D) Distribution plots are shown for both the qPCR and quantile normalized RNA-Seq read counts. Only genes assayed by qPCR are shown. Heterogeneous genes are shown in blue. i) qPCR. ii) Normalised RNA-Seq read counts.

Figure S4, related to Figure 4; SLAM Sca^{lo} and SLAM Sca^{hi} populations predominantly express other HSC markers.

A) Sorting strategy used to isolate SLAM Sca^{lo} and SLAM Sca^{hi} cells. CD48^{-}CD150^{+} were selected (left hand panel) and the cells staining positively for Sca-1 were divided into low and high expression (right hand panel). FACs plot as in Figure 4a, shown here for reference. B) Histograms showing the MFI for each additional HSC marker (CD34 (negative), c-Kit

(positive), FLT3 (negative), and EPCR (positive)) are shown for both SLAM Sca^{lo} (upper, blue) and SLAM Sca^{hi} (lower, blue) cells. The blue populations are overlaid onto the red, the latter showing the cell distribution of each marker for the whole bone marrow. Notably, both SLAM Sca^{lo} and SLAM Sca^{hi} express all HSC markers as expected.

Figure S5, related to Figure 5; Linking molecular states with functional outcomes.

A) Sca-1 expression overlaid over the subset of sequenced cells shown in Figure 5d. The cells in the lower left-hand cluster have significantly higher Sca-1 expression and significantly higher MolO score compared to the cells in the upper right-hand cluster. Axes are in arbitrary units. B) Joint t-SNE representation of RNA-Seq data and functional data coloured according to SuMO score. Axes are in arbitrary units. C) t-SNE for 4 of the 5 patients, showing distinct clusters of mammary luminal progenitor cells defined by hierarchical clustering in the 2D t-SNE space; two of the bottom 3 clusters (5 and 6) are enriched for colony-forming cells. D) t-SNE with overlay of luminal colony-forming. Red circles indicate successful colony formation, blue circles did not form colonies. (Over-representation of colony presence in clusters 5 and 6, p=0.00001). E) Bar graph showing the colony formation across all cells and the post-enrichment colony formation (bottom clusters only) in individual patient samples (A-D). A 5th sample was not enriched using the clustering tool. F) Differential expression between enriched (5 and 6) and non-enriched clusters (1, 2, 3 and 4) of the t-SNE map for the markers used to isolate the luminal progenitors.

Table S1, related to Figure 1; Fluidigm Taqman Assays.

A summary of the TaqMan assays used for single cell gene expression analysis.

Table S2, related to Figure 3; Differentially expressed genes as identified by scRNA-Seq.

The 4533 genes identified by scRNA-Seq to be differentially regulated between individual single cells and for which the biological variability exceeded technical variability.

Table S3, related to Figure 3; Differentially expressed genes ranked based on their MolO score.

Top 500 ranked scRNA-Seq genes by the MolO score. Top negatively correlated genes (NoMO). Top positively correlated genes (MolO).

Table S4, related to Figure 5; Differentially expressed genes based on their SuMO score.

The top 500 ranked scRNA-Seq genes by the SuMO score. Top negatively correlated genes (non-SuMO). Top positively correlated genes (SuMO).

Table S1: Fluidigm assays

Gene name	Assay ID
Bptf	Mm01251151_m1
Cbfa2t3h	Mm00486780_m1
Cdkn2a	Mm00494449_m1
Csf1r	Mm01266652_m1
Dnmt3a	Mm00432881_m1
Egfl7	Mm00618004_m1
Eif2b1	Mm01199614_m1
Erg	Mm01214246_m1
Ets1	Mm01175819_m1
Ets2	Mm00468977_m1
Etv6	Mm01261325_m1
Fli-1	Mm00484409_m1
Gata1	Mm00484678_m1
Gata2	Mm00492300_m1
Gata3	Mm00484683_m1
Gfi1	Mm00515855_m1
Gfi1b	Mm00492318_m1
Hhex	Mm00433954_m1
Hoxa5	Mm00439362_m1
Hoxa9	Mm00439364_m1
Hoxb4	Mm00657964_m1
Ikzf1	Mm01187882_m1
Itga2b	Mm00439768_m1
kit	Mm00445212_m1
Ldb1	Mm00440156_m1
Lmo2	Mm01281680_m1
Lyl1	Mm01247198_m1
Mecom	Mm01289155_m1
Meis1	Mm00487659_m1
Mitf	Mm01182480_m1
Mpl	Mm00440310_m1
Myb	Mm00501741_m1
Nfe2	Mm00801891_m1
Nkx2-3	Mm01199403_m1
Notch1	Mm00435249_m1
Pbx1	Mm04207617_m1
Polr2a	Mm00839493_m1
Prdm16	Mm00712556_m1
Procr	Mm00440993_mH
Runx1	Mm01213405_m1
Spi1	Mm00488142_m1
Sh2b3	Mm00493156_m1
Smarcc1	Mm00486224_m1
Tal1	Mm01187033_m1

Tcf7	Mm00493445_m1
Tet2	Mm00524395_m1
UBC	Mm01201237_m1
Vwf	Mm00550376_m1

Supplemental Experimental Procedures

Purification of Stem and Progenitor Cells.

Suspensions of BM cells from the femurs, tibiae and iliac crest of 8–12-week-old C57BL/6 mice were isolated and depleted of red blood cells by an ammonium chloride lysis step (STEMCELL Technologies, STEMCELL)). HSCs were isolated using the following antibodies: CD45-FITC or APC-Cy7 (Clone 30-F11 Biolegend), EPCR-PE (Clone RMEPCR1560**,** STEMCELL), CD150-Pacific Blue or PE-Cy7 (Clone TC15-12F12.2, Biolegend), CD48-APC (Clone HM48-1, Biolegend), Sca-1-Pacific Blue or PE (Clone E13-161.7, Biolegend), FLT3-PE or PE-Cy5 (Clone A2F10, eBioscience), CD34-FITC (Clone RAM34, BD Biosciences), c-kit APC-Cy7 (Clone 2B8, Biolegend), and a panel of lineage markers (Hematopoietic Progenitor Enrichment Cocktail, STEMCELL) plus streptavidin-V500 (BD Biosciences). The side population (SP) cells were isolated according to the detailed protocol found at https://www.bcm.edu/research/labs/goodell/index.cfm?pmid=20017 and as previously published (Challen et al., 2012). The Hoechst dye was obtained from Sigma and used at a final concentration of 5 μg/ml. Cells were sorted using a BD Influx sorter equipped with 355 nm, 405 nm, 488 nm, 561 nm, and 640 nm lasers. For single cell gene expression assays, cells were sorted into individual wells of 96 well PCR plates using a modified plate holder for increased stability. For single cell transplantation and *in vitro* assays, cells were sorted into individual wells of a U-bottom 96 well plate. For progenitor colony forming cell assays and 10-cell transplantation assays, cells were sorted into 1.5 ml tubes containing serum free media.

Progenitor Cell Assays

500 CMPs (Lin^{-}c-kit^{+}Sca-1^{-}CD34^{+}FcγR^{low}), MEPs (Lin^{-}c-kit^{+}Sca-1^{-}CD34^{-}FcγR^{low}), and GMPs (Lin^{-}c-kit^{+}Sca-1^{-}CD34^{+}FcγR^{hi}) were isolated into 1.5ml Eppendorf tubes containing serum-free medium. Cells were then divided into a high concentration fraction (~450 cells) and a low concentration fraction (~45 cells) and placed into a semi-solid medium containing growth factors to support growth of all myeloid colony types (MC3434, STEMCELL). Both high and low concentration fractions were spread across 3 individual wells of a 6 well plate and counted after 10 and 14 days of culture. Single LMPPs (Lin^{-}c-kit^{+}Sca-1^{+}CD34^{+}FLT3^{+}) were sorted into individual wells containing OP9 cells supplemented with 100 ng/ml IL-7 and 50ng/ml FLT-3. All wells were harvested at day 28 and analyzed for the presence of B- (defined as B220+) and myeloid (Ly6g and/or Mac1) cells.

Single HSC Cultures

SLAM Sca^{hi} and SLAM Sca^{lo} HSCs were sorted and cultured in STEMSPAN medium containing 300 ng/ml SCF, 20 ng/ml IL-11, Glutamine, Penicillin, Streptomycin, β-2-mercaptoethanol and 10% FCS as described previously (Kent et al., 2008; Kent et al., 2013). After 24 hours, wells were scored for the presence of a single cell and counted each day to track the clonal growth of individual cells. For the immunophenotyping studies, clones were individually stained and assessed for the expression of Sca-1, c-Kit, and a panel of lineage markers along with 7-Aminoactinomycin D (7AAD, Invitrogen) to mark dead cells.

Clone Size Calculations and Antibody Information for *In Vitro* Cultures

When the clones began to appear, they were estimated to be small (50-5000 cells), medium (5000-20,000 cells), or large (20,000 or more cells). No clones had fewer than 50 cells. 10-

day clones were stained with biotinylated lineage marker antibodies (Haematopoietic Progenitor Enrichment Cocktail, STEMCELL), c-Kit-APC (BD) and Sca-1-Pacific Blue (Biolegend). To enumerate cells, a defined number of fluorescent beads (Trucount Control Beads, BD) were added to each well and each sample was back-calculated to the proportion of the total that were run through the cytometer. Small clones were not able to be assessed individually by flow cytometry and were pooled – the % of KSL cells was greater than 95%. Flow cytometry was performed on an LSRII Fortessa (BD) and all data were analyzed using Flowjo (Treestar, USA).

Single-Cell Gene Expression Analysis.

Single-cell gene expression analysis was performed as described previously (Moignard et al., 2013). Briefly, single cells were sorted by FACS directly into individual wells of 96-well plates containing lysis and preamplification mix. Reverse transcription and specific target amplification were performed in the same plates 24 hours after sorting. cDNA was diluted 1:5 with TE before qPCR on the BioMark HD. For the qPCR, Taqman assays (Life technologies) and cDNA samples were then loaded into a 48.48 Dynamic Array (Fluidigm), and then transferred to the BioMark HD for qPCR.

Bioinformatic Analysis of Single-Cell Gene Expression Data.

Single-cell expression data were collected using the Fluidigm Data Collection software. ΔCt values were calculated as previously described (Guo et al., 2010) by cell-wise normalization to the mean expression level of two housekeeping genes (Ubc and Polr2a). Briefly, Ct values were subtracted from the limit of detection of the BioMark (Ct 27) (Guo et al., 2010),

followed by subtraction of the mean Ct value of Ubc and Polr2a for each cell. The ΔCt value for genes that were not expressed was set to be 3.5 cycles more than the lowest Ct value per gene. All housekeepers, Cdkn2a and Egfl7 were removed from the dataset for downstream analysis. Cdkn2a was not expressed in any of the cell types and Egfl7 assay experienced technical issues. Hierarchical clustering was performed in R (www.r-project.org) using the hclust package and heatmap.2 from the gplots package using Spearman rank correlations and ward linkage.

t-SNE was performed in Matlab (Mathworks Inc., Natick, USA) using the Matlab implementation (http://homepage.tudelft.nl/19j49/t-SNE.html) with standard settings.

We identified MolO cells based on a weighting matrix defined by repopulation probabilities and the 2D t-SNE representation of the data. As cells with similar gene expression patterns are located in close proximity in the 2D t-SNE representation of the data, we aimed to identify a contiguous region in the t-SNE plot where cells within the region locally fulfil the mixture constraint derived from the weighting matrix. We therefore iteratively assessed the local neighbourhoods of all cells for molecular overlap and tested whether within each neighbourhood no progenitors were present and the fraction of each HSC subtype exceeded 80% of its respective weight. We introduced the leeway factor of 80% to allow for some sampling variation. Robustness analysis was performed additionally which assessed the leeway factors of 70 and 90 percent (Figure S2b and S2c). If both conditions were met, the cell was classified as MolO core cell and all cells within its neighbourhood were classified as MolO cells. We performed a grid search to determine the neighbourhood size maximising the number of density-reachable MolO core points (Ester, 1996).

Random forests were trained on the normalised Ct values of the set of genes which were assayed by single-cell gene expression and variable above technical noise in scRNA-Seq.

Training was performed on all cells from sorting strategy HSC1 and generalizability was quantified using 10-fold cross-validation (Figure S3c). For prediction of the sequenced cells, read counts had to be transformed to the same scale as normalised Ct values. As both sets of cells originated from the same sorting strategy we reasoned that the distributions of expression values of variable genes should be similar for the qPCR and the RNA-Seq data and performed quantile normalisation for the respective subsets of cells expressing each variable gene (Figure S3d). Training and testing of the classifier was performed in python 2.7 using the sklearn library.

Single-Cell RNA-Seq

Single cell RNA-Seq analysis was performed as described previously (Picelli et al., 2014). Briefly, single cells were sorted by FACS directly into individual wells of a 96-well plate containing lysis buffer. Reverse transcription and PCR amplification were performed in the same plates. The resulting PCR products were purified, the quality of the cDNA library verified and libraries were prepared using the Illumina Nextera XT DNA preparation kit. Pooled libraries were then run on the Illumina Hi-Seq 2500. Files were demultiplexed and reads aligned to the mouse genome (mm9) using STAR (Dobin et al., 2013). HTSeq (Anders, 2014) was run on all samples to assign mapped reads to genes, using Ensembl genes as a reference. Several QC steps were implicated, such that a single cell was required to have >500k unique counts mapping to annotated features, <10% of the counts mapped to mitochondria DNA and >4000 genes detected. Mapped reads were normalised using size factors as described in Brenencke et al (Brennecke et al., 2013). To estimate technical noise we further followed Brenencke et al (Brennecke et al., 2013) and fitted the relation between mean read counts and squared coefficient of variation using the ERCC spike-ins (Life

technologies) (Figure 3a i). Genes for which the squared coefficient of variation exceeded technical noise were considered variable. To analyse the robustness of PC1, we sub-sampled the data by repeating the PCA based on a random subset of 86 cells. We then quantified the consistency of the PCA representations by projecting the 5 outlier cells onto the new principal components, and calculated the correlation between the sub-sampled PC1 and PC1 from all cells. We repeated this procedure 500 times and the correlation coefficient was always greater than 0.98. If we omit the 5 outlier cells from the original PCA, the correlation coefficient was 0.983, giving good confidence in the clusters described.

Transplantation of Haematopoietic stem cells

10-cell transplantations were performed in CD45.1 lethally irradiated C57Bl/6 recipients along with 250,000 helper cells from the spleen of CD45.1/.2 mouse. All single cell transplantations were performed by standard intravenous tail vein injection of sublethally irradiated Ly5-congenic adult W41/W41 mice as previously described (Dykstra et al., 2007). Peripheral blood samples were collected from the tail vein of some mice at 4 weeks and all mice at 8, 16, and 24 weeks after transplantation and red blood cells lysed using ammonium chloride (Stemcell technologies, Stemcell). All samples were stained with the following antibody panel: Ly6g-Pacific Blue (Clone 1A8, Biolegend), Mac1-FITC (Clone M1/70, Biolegend), CD3e-PE (Clone 17A2, BD), B220-APC (Clone RA3-6B2, eBioscience), CD45.1-Alexa700 (Clone A20, Biolegend) and CD45.2-APC-Cy7 (Clone 104, BD)). Donor and recipient cells were distinguished by their expression of CD45.1 or CD45.2 and any double positive CD45.1 and CD45.2 events were excluded from the analysis of donor contributions to specific whole blood cell (WBC) subsets (leftover helper cells and/or doublets). Transplanted cells from which at least 1% of the WBCs and were derived at 16

and/or 24 weeks after transplantation were considered to be repopulated with long-term reconstituting cells. HSCs were further discriminated according to previously described high (alpha or beta) or low (gamma or delta) ratios of their proportional contributions to the GM, B- and T-cell subsets of the circulating WBCs assessed at 16 weeks after transplantation (Dykstra et al., 2007). Flow cytometry was performed on an LSRII Fortessa (BD) and all data were analyzed using Flowjo (Treestar, USA).

Isolation and assessment of mammary progenitors

All primary human material was derived from 5 reduction mammoplasties at Addenbrooke's Hospital, Cambridge, UK, under full informed consent and in accordance with the National Research Ethics Service, Cambridgeshire 2 Research Ethics Committee approval (08/H0308/178) as part of the Adult Breast Stem Cell Study. All tissue donors had no previous history of cancer and were premenopausal (ages 20 to 23). Mammary tissue was dissociated to single cell suspensions as previously described (Eirew et al., 2010). Single cell suspensions of human mammary cells were treated to detect the enzyme activity of aldehyde dehydrogenase (ALDH) using the Aldefluor Kit (StemCell Technologies) as per the manufacturer's instructions. The cells were incubated with the following primary antibodies: CD49f-PE/Cy7 (clone: GoH3), epithelial cell adhesion molecule (EpCAM)-PE (clone: 9C4), and CD45-APC-Cy7 (clone: HI30), CD31-APC-Cy7 (clone: WM-59) (collectively known as Lin-APC-Cy7), as well as 4',6-diamidino-2-phenylindole (DAPI) for viability. Hank's balanced salt solution supplemented with 2% FBS (Gibco) was used as the diluent for all antibody incubation and washing steps. Cells were sorted using a BD Influx. Single-stained control cells were used to perform compensation manually and gates were set in reference to fluorescence minus-one-controls. The ALDH+ gate was set in reference to control

populations incubated with the ALDH inhibitor, DEAB in addition to Aldefluor. Luminal progenitor populations were seeded single cell into 96 well plates with 1×10^4 irradiated NIH-3T3 feeder cells. The cultures were maintained in Human EpiCult-B (StemCell Technologies) supplemented with 5% FBS (StemCell Technologies) and 50 μg/ml gentamicin and maintained for 10 to 12 days. Colony presence was scored under a microscope.

Supplemental References

Anders, S., Pyl, P, T., and Huber, W. (2014). HTSeq - A Python framework to work with high-throughput sequencing data. bioRxiv.

Brennecke, P., Anders, S., Kim, J.K., Kolodziejczyk, A.A., Zhang, X., Proserpio, V., Baying, B., Benes, V., Teichmann, S.A., Marioni, J.C., *et al.* (2013). Accounting for technical noise in single-cell RNA-seq experiments. Nat Methods *10*, 1093-1095.

Challen, G.A., Sun, D., Jeong, M., Luo, M., Jelinek, J., Berg, J.S., Bock, C., Vasanthakumar, A., Gu, H., Xi, Y., *et al.* (2012). Dnmt3a is essential for hematopoietic stem cell differentiation. Nat Genet *44*, 23-31.

Dobin, A., Davis, C.A., Schlesinger, F., Drenkow, J., Zaleski, C., Jha, S., Batut, P., Chaisson, M., and Gingeras, T.R. (2013). STAR: ultrafast universal RNA-seq aligner. Bioinformatics *29*, 15-21.

Dykstra, B., Kent, D., Bowie, M., McCaffrey, L., Hamilton, M., Lyons, K., Lee, S.J., Brinkman, R., and Eaves, C. (2007). Long-term propagation of distinct hematopoietic differentiation programs in vivo. Cell Stem Cell *1*, 218-229.

Eirew, P., Stingl, J., and Eaves, C.J. (2010). Quantitation of human mammary epithelial stem cells with in vivo regenerative properties using a subrenal capsule xenotransplantation assay. Nat Protoc *5*, 1945-1956.

Ester, M., Kriegel, H, -P,. Sander, J, Xu, X. (1996). A density-based algorithm for discovering clusters in large spatial database with noise. Proceedings of Second International Conference on Knowledge Discovery and Data Mining, 226-231.

Guo, G., Huss, M., Tong, G.Q., Wang, C., Li Sun, L., Clarke, N.D., and Robson, P. (2010). Resolution of cell fate decisions revealed by single-cell gene expression analysis from zygote to blastocyst. Dev Cell *18*, 675-685.

Kent, D.G., Dykstra, B.J., Cheyne, J., Ma, E., and Eaves, C.J. (2008). Steel factor coordinately regulates the molecular signature and biologic function of hematopoietic stem cells. Blood *112*, 560-567.

Kent, D.G., Li, J., Tanna, H., Fink, J., Kirschner, K., Pask, D.C., Silber, Y., Hamilton, T.L., Sneade, R., Simons, B.D., *et al.* (2013). Self-renewal of single mouse hematopoietic stem cells is reduced by JAK2V617F without compromising progenitor cell expansion. PLoS Biol *11*, e1001576.

Moignard, V., Macaulay, I.C., Swiers, G., Buettner, F., Schutte, J., Calero-Nieto, F.J., Kinston, S., Joshi, A., Hannah, R., Theis, F.J., *et al.* (2013). Characterization of transcriptional networks in blood stem and progenitor cells using high-throughput single-cell gene expression analysis. Nat Cell Biol *15*, 363-372.

Picelli, S., Faridani, O.R., Bjorklund, A.K., Winberg, G., Sagasser, S., and Sandberg, R. (2014). Full-length RNA-seq from single cells using Smart-seq2. Nat Protoc *9*, 171-181.
